# Hugan Tablets Alleviate Alcoholic Liver Injury by Modulating Hepatic Glutathione Metabolism and PPARγ/NRF2/GPX4-Related Antioxidant Defense

**DOI:** 10.3390/ph19071007

**Published:** 2026-06-29

**Authors:** Ruishu Chen, Miao Li, Huajinzi Li, Xiaoyan Gao

**Affiliations:** School of Chinese Materia Medica, Beijing University of Chinese Medicine, Beijing 102488, China; ccccrs2002@163.com (R.C.); lm15731876830@163.com (M.L.); lhjz321@163.com (H.L.)

**Keywords:** Hugan tablets, alcoholic liver injury, glutathione metabolism, metabolomics, multi-omics, transcriptomics

## Abstract

**Background**: Hugan tablets (HGP) are a commercially available traditional Chinese medicine preparation used for liver disorders, but the mechanisms underlying their effects on alcoholic liver injury (ALI) remain incompletely understood. This study investigated the hepatoprotective effects and potential mechanisms of HGP in ALI. **Methods**: An ALI mouse model was established using a Lieber–DeCarli ethanol liquid diet. The effects of HGP were evaluated using biochemical and histopathological assessments, followed by integrated liver and serum metabolomics, liver transcriptomics, and ELISA-based protein validation. **Results**: HGP alleviated alcohol-induced liver injury, hepatic lipid accumulation, oxidative stress, and inflammatory responses. Integrated multi-omics analyses indicated that HGP treatment was associated with changes in hepatic glutathione metabolism, PPAR signaling, and antioxidant-related processes. ELISA validation showed increased measured concentrations in liver homogenate supernatants of PPARγ, NRF2, GCL, and GPX4 following HGP treatment. These findings support the potential involvement of a PPARγ/NRF2/GPX4-related antioxidant network. **Conclusions**: HGP alleviated ALI in mice, and its effects may be associated with modulation of hepatic glutathione metabolism and a PPARγ/NRF2/GPX4-related antioxidant network. These findings provide experimental evidence for the potential use of HGP in alcohol-induced liver injury.

## 1. Introduction

Chronic and excessive alcohol consumption can lead to alcohol-associated liver disease (ALD), which encompasses a broad clinical spectrum ranging from hepatic steatosis and alcohol-associated hepatitis to cirrhosis and hepatocellular carcinoma. Approximately 90–100% of chronic heavy drinkers develop alcoholic fatty liver, and 10–20% of these individuals further progress to advanced ALD, such as cirrhosis or hepatocellular carcinoma [1]. According to the 2023 Global Burden of Liver Disease Report, alcohol accounts for nearly 60% of cirrhosis cases in Europe, North America, and Latin America [2], with alcohol-related liver cirrhosis contributing to as many as 42% of cirrhosis-related deaths [3]. ALD imposes a considerable burden on human health. Current therapeutic options remain limited, and alcohol abstinence remains the most effective intervention [4,5]. Therefore, there is an urgent clinical need to elucidate the pathological mechanisms underlying ALD and to identify safe and effective therapeutic strategies.

Alcohol-induced liver injury represents a central pathological component of the broader ALD spectrum and is closely associated with an imbalance between oxidative stress and antioxidant defense [6]. Ethanol metabolism, particularly through cytochrome P450 family 2 subfamily E member 1 (CYP2E1)-related processes, promotes reactive oxygen species production and contributes to the depletion of glutathione, thereby increasing hepatocellular oxidative damage [7]. Studies have shown that, as an adaptive response to oxidative stress induced by CYP2E1, nuclear factor erythroid 2-related factor 2 (NRF2), which regulates GSH level, is upregulated following chronic alcohol exposure [8]. NRF2 is a transcription factor that is highly sensitive to oxidative stress. By sensing changes in the intracellular redox state, it regulates the expression of enzymes involved in glutathione metabolism, including glutamate-cysteine ligase (GCL) and glutathione peroxidase 4 (GPX4), thereby protecting cells from oxidative damage. Previous studies have shown that activation of the NRF2-GPX4 axis can ameliorate ethanol-induced hepatocyte injury, and NRF2 can directly enhance GPX4 expression by binding to antioxidant response elements (AREs) in stress-responsive genes [9]. Furthermore, oxidative stress resulting from alcohol metabolism also disrupts lipid homeostasis. CYP2E1-dependent ROS generation has been shown to inhibit the peroxisome proliferator-activated receptor (PPAR) signaling pathway and downregulate the expression of its target gene, acyl-CoA oxidase, thereby impairing fatty acid β-oxidation and exacerbating triglyceride (TG) accumulation in hepatocytes [10]. Notably, there is an interplay between NRF2 and the PPARγ signaling pathway. PPARγ can influence the expression of the *Nfe2l2* gene, thereby playing a role in regulating antioxidant responses and metabolic balance [11,12,13]. In summary, coordinated responses involving PPARγ, NRF2, and GPX4 may constitute a key network in defending against ALI.

Hugan Tablets (HGP) is a traditional Chinese patent medicine developed from the classical prescriptions Xiaochaihu Decoction and Yinchenhao Decoction, both of which were originally recorded in the Treatise on Febrile Diseases. HGP has been widely used in China for the management of liver-related disorders. The formula of HGP is composed of Bupleuri Radix (derived from *Bupleurum chinense* DC. or *Bupleurum scorzonerifolium* Willd.), Artemisiae Scopariae Herba (derived from *Artemisia capillaris* Thunb.), Schisandrae Chinensis Fructus (derived from *Schisandra chinensis* (Turcz.) Baill.), Isatidis Radix (derived from *Isatis tinctoria* L.), Pulvis Fellis Suis (derived from *Sus scrofa domestica* Brisson), and mung bean (derived from *Vigna radiata* (L.) R. Wilczek.). HGP is commercially available in China and has been used in the management of chronic hepatitis and early-stage liver cirrhosis [14,15]. Our previous studies characterized the chemical constituents and candidate bioactive components of HGP associated with its protective effects against alcohol-induced liver injury. Surface plasmon resonance (SPR) affinity analysis was employed, along with in vitro and in vivo experiments, to validate the bioactivity of the candidate compounds, providing a reference for the clinical application of HGP in the treatment of alcoholic liver disease [16,17]. However, its relationship with specific antioxidant regulators remains unclear. Given the established roles of PPARγ, NRF2, and GPX4 in metabolic and antioxidant responses, we hypothesized that coordinated changes involving these proteins may contribute to the hepatoprotective effects of HGP against alcohol-induced liver injury.

Given the complex nature of ALD, experimental models are commonly used to investigate potential therapeutic interventions and underlying mechanisms. In this study, a mouse model of ALI induced by a Lieber-DeCarli ethanol liquid diet was employed to evaluate the therapeutic efficacy of HGP against ALI, which reflects key pathological features of ALD [18]. This model was used to evaluate the hepatoprotective effects of HGP. Potential pathways involved in the action of HGP against ALI were identified through database screening and literature mining. On this basis, integrated metabolomic and transcriptomic analyses were conducted to further explore the underlying mechanisms by which HGP ameliorates ALI.

## 2. Results

### 2.1. HGP Alleviates Liver Injury in ALI Mice

**Figure 1:**
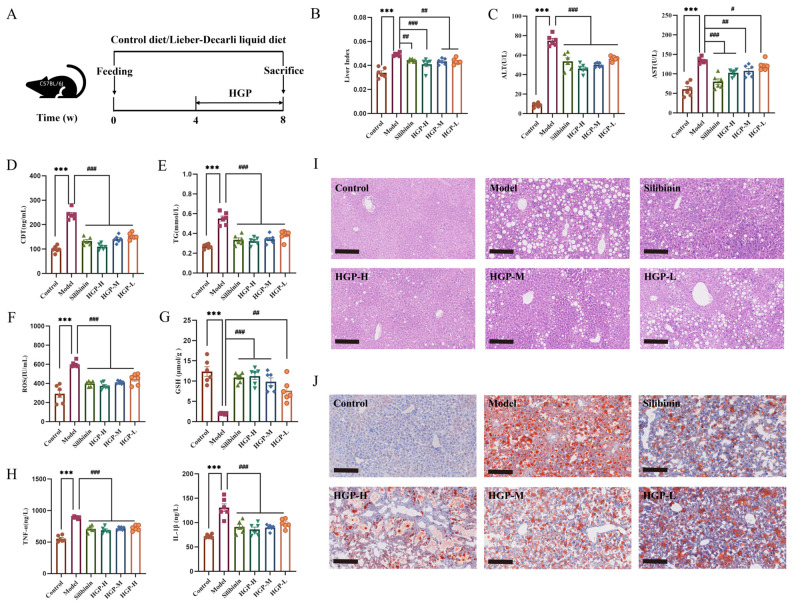
Pharmacodynamic effects of HGP in mice with alcoholic liver injury. (**A**) Experimental design and treatment schedule. Mice were assigned to Control, Model, Silibinin, HGP-H, HGP-M, and HGP-L groups. (**B**) Liver index. (**C**) Serum ALT and AST levels. (**D**) Serum CDT level. **(E)** Hepatic TG level. (**F**) Hepatic ROS level. (**G**) Hepatic GSH level. (**H**) Hepatic TNF-α and IL-1β levels. (**I**) Representative H&E-stained liver sections. (**J**) Representative Oil Red O-stained liver sections. Data are presented as mean ± SD (*n* = 6) and analyzed by one-way ANOVA followed by Tukey’s multiple-comparison test. Histological images were acquired at 20× magnification (scale bar = 100 μm). *** *p* < 0.001 vs. Control; # *p* < 0.05, ## *p* < 0.01, ### *p* < 0.001 vs. Model.

First, the protective effect of HGP against ALI in mice was validated. The animal experimental procedure is shown in Figure 1A. At the end of the experiment, compared with the control group, the model group showed a significant increase in final body weight and a higher liver wet weight, although the difference in liver wet weight was not statistically significant. Final body weight was significantly decreased in the silibinin and HGP-H groups compared with the model group, while no significant changes were observed in the HGP-L and HGP-M groups. Liver wet weight showed a similar trend, with a significant reduction only in the HGP-H group (Figure S1). Consistently, HGP reduced the alcohol-induced increase in liver index (Figure 1B). Alanine aminotransferase (ALT) and aspartate aminotransferase (AST) are commonly used biochemical indicators of hepatocellular injury, whereas carbohydrate-deficient transferrin (CDT) is a biomarker associated with chronic or sustained alcohol exposure. Compared with the control group, the model group showed significantly increased serum ALT, AST, and CDT levels. The elevations in ALT and AST, together with hepatic lipid accumulation and histopathological changes, confirmed successful establishment of the ALI model. CDT was included as a supplementary marker of sustained alcohol exposure. Administration of silibinin and various doses of HGP effectively attenuated the increases in serum ALT, AST, and CDT levels (Figure 1C,D). Chronic and excessive alcohol consumption can lead to intrahepatic lipid accumulation, oxidative stress, and inflammation. Administration of HGP significantly ameliorated hepatic TG accumulation (Figure 1E), oxidative stress levels (Figure 1F,G), and the release of inflammatory cytokines (Figure 1H). Hematoxylin and eosin (H&E) staining and Oil Red O staining results (Figure 1I,J) showed that HGP alleviated hepatocyte necrosis, steatosis, and inflammatory cell infiltration in the liver tissue of ALI mice, and also improved alcohol-induced lipid accumulation within hepatocytes. Among the tested doses, the high-dose HGP group showed relatively larger improvements in several measured endpoints and was therefore selected for the subsequent exploratory omics analyses.

### 2.2. Exploratory Network Pharmacology Analysis of HGP Intervention in ALI

**Figure 2:**
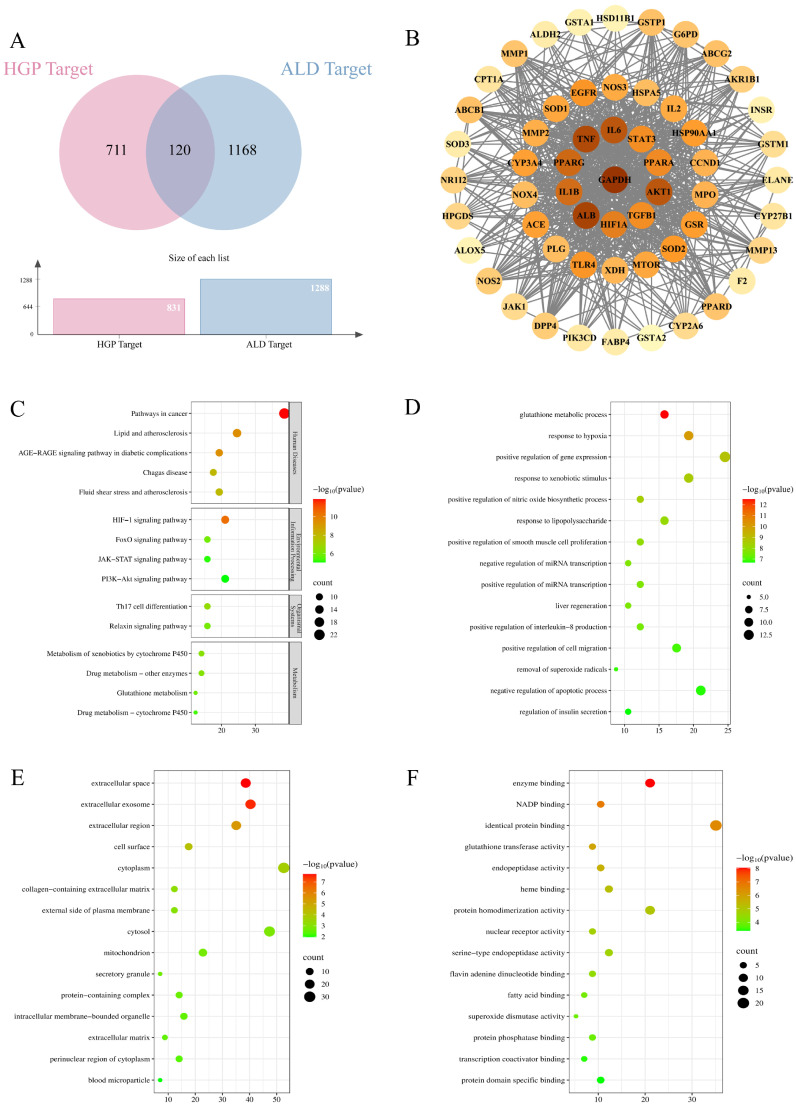
Exploratory network pharmacology analysis of HGP intervention in ALI. (**A**): Venn diagram showing the intersection between HGP-related targets and alcohol-related liver disease-associated targets; (**B**): PPI network of core predicted candidate targets(The darker the color, the greater the degree of connection of the protein.); (**C**): KEGG enrichment analysis result; (**D**): Gene Ontology biological process (GO BP) enrichment analysis result; (**E**): Gene Ontology cellular component (GO CC) enrichment analysis result; (**F**): Gene Ontology molecular function (GO MF) enrichment analysis result.

Network pharmacology was performed as an exploratory computational analysis to provide complementary information on potential biological processes related to HGP intervention in ALI. Potential targets of the chemical components and in vivo metabolites of specific ingredients in HGP were retrieved from the Swiss Target Prediction and ETCM databases. After removing duplicates, a total of 831 targets were identified. Alcohol-related liver disease-associated targets were obtained from the GeneCards and OMIM databases, yielding 1288 targets after deduplication. The intersection between the alcohol-related liver disease-associated targets and HGP-related targets yielded 120 common predicted candidate targets (Figure 2A). The 120 common targets were imported into the STRING platform for protein–protein interaction (PPI) analysis, resulting in 116 nodes and 911 edges, with an average degree of 15.2 and an average local clustering coefficient of 0.592. The PPI results were then imported into Cytoscape v3.10.3 software, and the degree centrality of all nodes was calculated through network analysis. The targets were ranked in descending order of degree values, and the top 57 targets with degree values above the median were selected as core predicted candidate targets and visualized (Figure 2B). Enrichment analysis of the 57 core predicted candidate targets identified by PPI was performed using the DAVID database. The enrichment results indicated that these targets were closely associated with oxidative stress-related pathways (Figure 2C–F), such as the glutathione metabolic process, response to hypoxia, superoxide radical removal, glutathione transferase activity, HIF-1 signaling pathway, FoxO signaling pathway, and PI3K-Akt signaling pathway, as well as inflammation-related pathways, including response to xenobiotic stimulus, response to lipopolysaccharide, JAK-STAT signaling pathway, and Th17 cell differentiation. These findings provided complementary information consistent with oxidative stress- and glutathione metabolism-related processes, which were further examined using metabolomic, transcriptomic, and experimental validation approaches.

### 2.3. HGP Treatment Is Associated with Changes in Metabolic Features Related to Glutathione Metabolism

**Figure 3:**
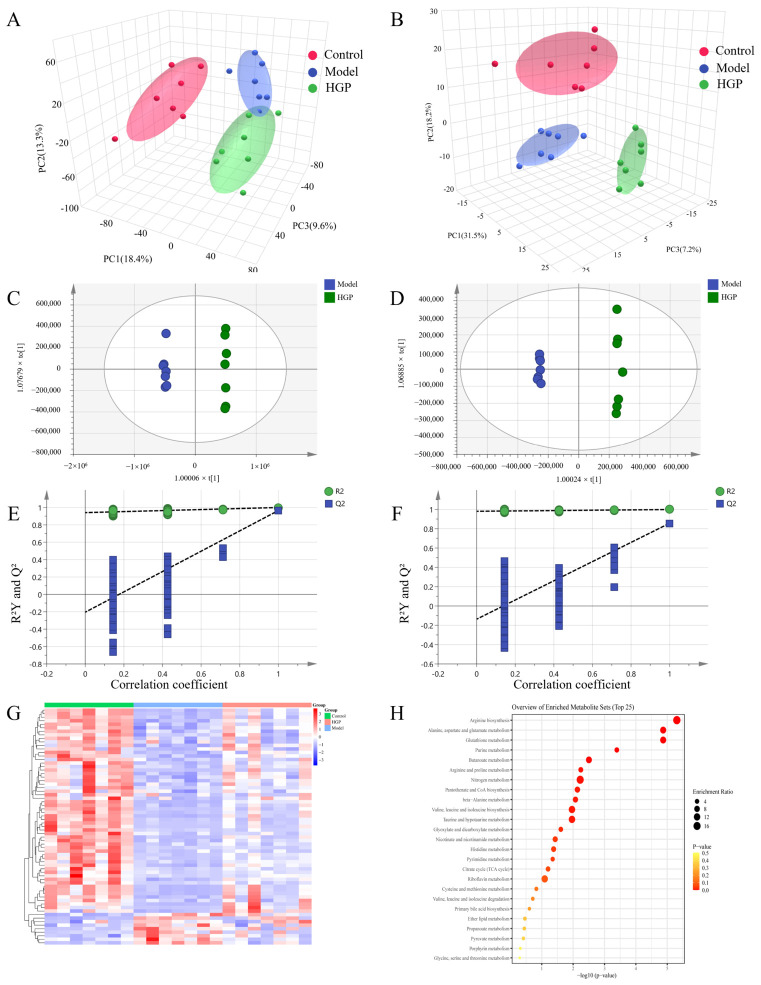
Hepatic non-targeted metabolomics results. (**A**): PCA score plot in positive ion mode; (**B**): PCA score plot in negative ion mode; (**C**): OPLS-DA score plot between the model and HGP groups in positive ion mode; (**D**): OPLS-DA score plot between the model and HGP groups in negative ion mode; (**E**): Permutation test of the OPLS-DA model between the model and HGP groups in positive ion mode; (**F**): Permutation test of the OPLS-DA model between the model and HGP groups in negative ion mode; (**G**): Relative abundances of annotated candidate metabolites showing counter-directional trends following HGP treatment in the control, model, and HGP groups; (**H**): KEGG metabolic pathway enrichment analysis results. (*n* = 7 per group).

The liver is the primary organ for alcohol metabolism and the pathological site of ALI. To investigate the effects of HGP on hepatic metabolism in ALI mice, non-targeted metabolomics analysis was performed on liver tissues. Principal component analysis (PCA) in positive and negative ion modes (Figure 3A,B) revealed clear separation among the control, model, and HGP groups, indicating that alcohol induced significant metabolic changes in the mouse liver, and that HGP treatment was associated with a shift in the hepatic metabolic profile toward that of the control group. Subsequently, orthogonal partial least squares discriminant analysis (OPLS-DA) was performed to further assess the metabolic differences between the model and HGP groups. In the positive and negative ion modes, the R^2^Y/Q^2^ values were 0.999/0.820 and 0.999/0.857, respectively, indicating good model fit and predictive ability. Model stability was further evaluated using 200 permutation tests. The Q^2^ intercept values were lower than 0, supporting the robustness of the models and suggesting no obvious overfitting (Figure 3C–F). Using variable importance in projection (VIP) > 1 and adjusted *p* value < 0.05 as screening criteria, 52 and 31 differential metabolites were annotated between the control and model groups in positive and negative ion modes, respectively. Among these, 42 and 25 putatively annotated candidate metabolites showed counter-directional trends following HGP treatment in positive and negative ion modes, respectively, as shown in Tables S1 and S2. The relative abundances of the 67 annotated metabolites across all samples were visualized using a heatmap (Figure 3G), further demonstrating that HGP effectively ameliorated the hepatic metabolic disturbances observed in ALI mice. Pathway enrichment analysis of these candidate metabolites was performed using MetaboAnalyst 6.0 (Figure 3H). Arginine biosynthesis; alanine, aspartate, and glutamate metabolism; glutathione metabolism; purine metabolism; and butanoate metabolism were identified as the most significantly perturbed metabolic pathways, which may be closely associated with the therapeutic effects of HGP.

**Figure 4:**
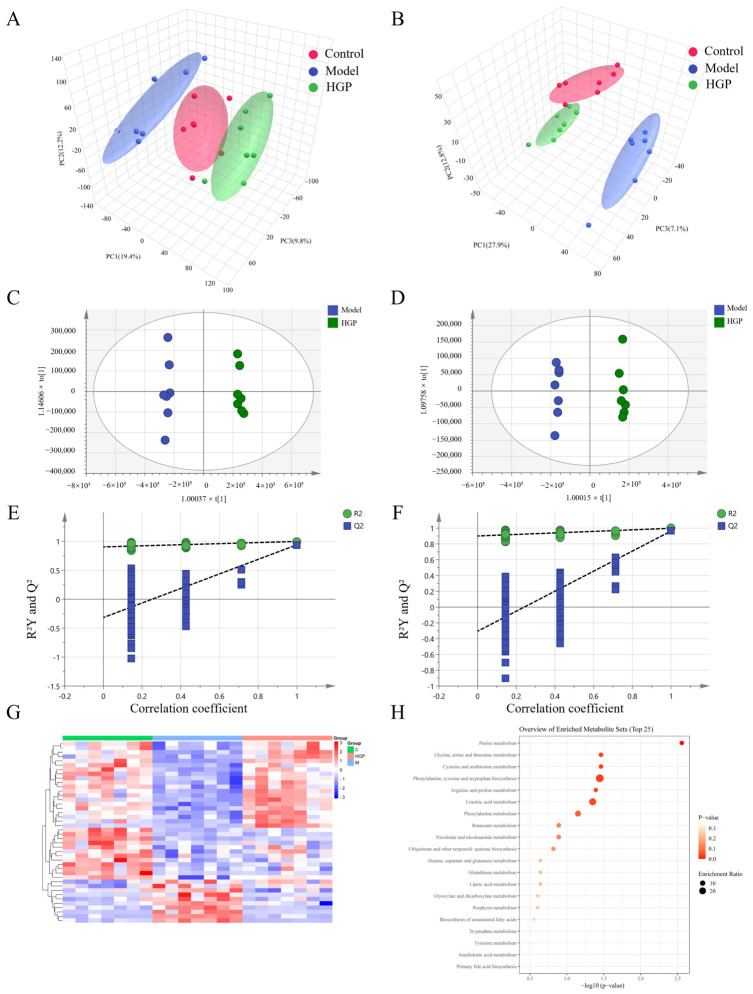
Serum non-targeted metabolomics results. (**A**): PCA score plot in positive ion mode; (**B**): PCA score plot in negative ion mode; (**C**): OPLS-DA score plot between the model and HGP groups in positive ion mode; (**D**): OPLS-DA score plot between the model and HGP groups in negative ion mode; (**E**): Permutation test of the OPLS-DA model between the model and HGP groups in positive ion mode; (**F**): Permutation test of the OPLS-DA model between the model and HGP groups in negative ion mode; (**G**): Relative abundances of annotated candidate metabolites showing counter-directional trends following HGP treatment in the control, model, and HGP groups; (**H**): KEGG metabolic pathway enrichment analysis results. (*n* = 7 per group).

Serum is a key component of the systemic circulatory system and comprehensively reflects the overall metabolic state of the organism. Serum non-targeted metabolomics can capture the impact of disease on systemic metabolism, providing more comprehensive information for elucidating drug mechanisms. To further clarify the mechanism by which HGP ameliorates the pathology of ALI, changes in serum metabolites were analyzed. The results showed that after HGP intervention, the serum metabolic profile of ALI mice was altered, with the PCA distribution in both positive and negative ion modes shifting closer to that of the control group (Figure 4A,B). OPLS-DA was subsequently performed to further assess the differences in serum metabolic profiles between the model and HGP groups. In the positive and negative ion modes, the R^2^Y/Q^2^ values were 0.994/0.902 and 0.997/0.962, respectively, indicating good model fit and predictive ability. Model stability was further evaluated using 200 permutation tests. The Q^2^ intercept values were lower than 0, supporting the robustness of the models and suggesting no obvious overfitting (Figure 4C–F). Using VIP > 1 and adjusted *p* value < 0.05 as screening criteria, HGP modulated a total of 44 metabolites (Tables S3 and S4). A total of 44 putatively annotated candidate metabolites met the screening criteria in the Control versus Model comparison and showed counter-directional trends following HGP treatment (Figure 4G). Further pathway analysis (Figure 4H) indicated that these candidates were associated with those observed in liver tissue, commonly enriching pathways such as butanoate metabolism; alanine, aspartate, and glutamate metabolism; glutathione metabolism; purine metabolism; glyoxylate and dicarboxylate metabolism; nicotinate and nicotinamide metabolism; and arginine and proline metabolism.

Analysis of the non-targeted metabolomics results from the liver and serum revealed that among the shared pathways in both tissues, glutathione metabolism; alanine, aspartate, and glutamate metabolism; arginine and proline metabolism; purine metabolism; nicotinate and nicotinamide metabolism; and glyoxylate and dicarboxylate metabolism all contribute to maintaining glutathione synthesis and function by providing precursors, regulating redox status, or participating in detoxification processes. Multiple annotated metabolites related to glutathione metabolism showed counter-directional trends following HGP treatment in the liver and serum. These findings suggest that glutathione metabolism may be involved in the metabolic response associated with HGP intervention.

### 2.4. HGP Modulates Glutathione Metabolism-Related Gene Expression in the Liver

All RNA samples met the predefined quality requirements for library construction and sequencing. Each library generated approximately 44.79 million raw reads on average, with Q30 values exceeding 95% and mapping rates above 93%, supporting the suitability of the sequencing data for subsequent transcriptomic analyses. Detailed quality-control results are provided in Supplementary Tables S5–S7 and Figures S2 and S3.

**Figure 5:**
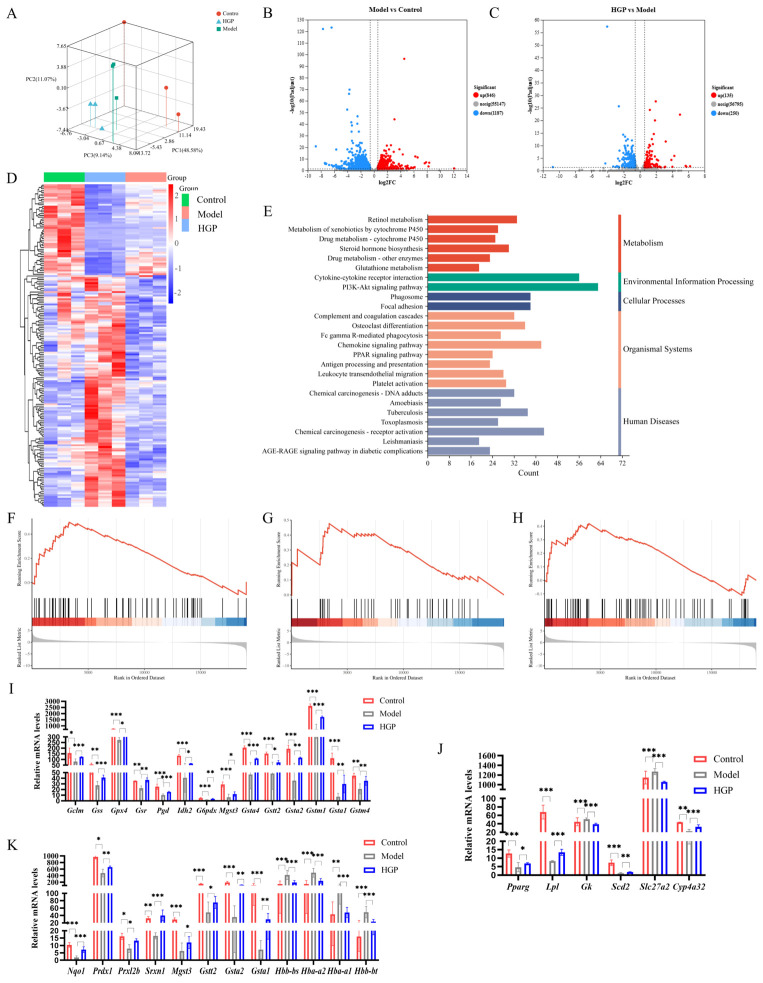
HGP intervention alters liver transcriptomic profiles in ALI mice. (**A**): PCA results; (**B**): Volcano plot of DEGs between the control and model groups; (**C**): Volcano plot of DEGs between the model and HGP groups; (**D**): Heatmap showing the expression patterns of DEGs showing opposite expression directions following HGP treatment; (**E**): KEGG enrichment analysis of DEGs showing opposite expression directions following HGP treatment; (**F**): GSEA results for all genes in the glutathione metabolism pathway between the model and HGP groups; (**G**): GSEA results for all genes in the PPAR signaling pathway between the model and HGP groups; (**H**): GSEA results for all genes in the antioxidant activity gene set between the model and HGP groups; (**I**–**K**): Expression levels of selected genes associated with glutathione metabolism, PPAR signaling, and antioxidant activity, respectively. Genes were selected based on a nominal *p* value < 0.05 in both the Model versus Control and HGP versus Model comparisons, opposite expression directions between the two comparisons, and membership in the corresponding gene sets. Data are presented as mean ± SD (*n* = 3 per group) and were analyzed by one-way ANOVA followed by Tukey’s multiple-comparison test. (compared with the model group, * *p* < 0.05, ** *p* < 0.01, *** *p* < 0.001).

To evaluate the overall hepatic transcriptional profiles, PCA was performed using the gene expression data from the three groups (Figure 5A). The samples from the control and model groups were clearly separated, indicating distinct hepatic transcriptional profiles between the two groups. The HGP samples were separated from the model samples, indicating that HGP treatment was associated with changes in the hepatic transcriptomic profile. Using a nominal *p* < 0.05, 4211 and 1783 genes showed nominal differential expression in the Model versus Control and HGP versus Model comparisons, respectively, with a similar number of upregulated and downregulated genes, each accounting for approximately half (Figure S4A,B). To identify genes with more robust expression changes, more stringent criteria of an adjusted *p* value < 0.05 and |log2FoldChange| > 0.585 were subsequently applied. Compared with the control group, the model group exhibited 2033 differentially expressed genes (DEGs), including 846 upregulated and 1187 downregulated DEGs (Figure 5B). A total of 181 DEGs met the stringent criteria in both comparisons and showed opposite expression directions. Among these, 51 DEGs were upregulated in the Model versus Control comparison and downregulated in the HGP versus Model comparison, whereas 130 DEGs were downregulated in the Model versus Control comparison and upregulated in the HGP versus Model comparison (Figure 5C,D and Table S8). These results indicate that alcohol induced substantial transcriptional changes in mice, and HGP treatment was associated with expression changes opposing a subset of the alcohol-induced alterations.

To further investigate the biological processes associated with alcohol exposure and HGP treatment, KEGG and GO enrichment analyses were performed using the selected DEGs. As shown in Figure S4C, KEGG analysis revealed that among the pathways significantly enriched by genes between the control and model groups, metabolism of cytochrome P450 was closely associated with alcohol metabolism. As a key member of the cytochrome P450 family, CYP2E1 plays a central role in alcohol metabolism through its metabolic characteristics and toxic mechanisms [19]. The PI3K-AKT signaling pathway [20], chemokine signaling pathway [21] and PPAR signaling pathway [22] were all closely associated with the onset and progression of alcohol-induced oxidative stress injury and inflammation. GO analysis results indicated that alcohol-induced hepatocyte injury was closely related to biological processes such as alcohol metabolic processes, immune cell activation involved in immune responses, fatty acid metabolism, reactive oxygen species metabolic processes, and regulation of inflammatory responses (Figure S4D–F).

Following HGP intervention, KEGG enrichment analysis revealed that the DEGs modulated by HGP were also enriched in glutathione metabolism and the PPAR signaling pathway (Figure 5E). Gene Set Enrichment Analysis (GSEA) was performed to explore the expression changes of all genes identified in the glutathione metabolism pathway. The results indicated that genes in the glutathione metabolism pathway (map00480) were predominantly upregulated (Figure 5F). Therefore, the expression changes of selected genes within the enriched gene set were further analyzed. Figure 5I reflects the expression levels of these genes in the control vs. model groups and the model vs. HGP groups. Clearly, alcohol exposure led to decreased expression of these genes, whereas HGP treatment was associated with changes in the opposite direction. The PPAR signaling pathway and glutathione metabolism pathway exhibit close interactions in oxidative stress, metabolic regulation, and inflammatory responses [23]. GSEA was therefore used to examine changes in the PPAR signaling pathway (map03320) following HGP treatment. GSEA indicated that compared with the model group, genes in the PPAR signaling pathway were predominantly upregulated in the HGP group (Figure 5G). Among them, *Pparg* showed a significant counter-directional expression change following HGP treatment (Figure 5J). The protein encoded by *Pparg* is peroxisome proliferator-activated receptor γ (PPARγ), which can upregulate the expression of various antioxidant enzyme genes, including *Gpx4*, thereby inhibiting excessive ROS in hepatocytes and alleviating oxidative stress. Collectively, these results suggest that HGP treatment was associated with coordinated changes in PPAR signaling-related genes.

Because genes annotated to antioxidant activity were significantly overrepresented among the DEGs in both the Model versus Control and HGP versus Model comparisons, and several of these genes showed opposite expression patterns between the two comparisons, we further examined antioxidant-related processes potentially associated with HGP intervention. GSEA showed that antioxidant activity (GO ID: 0016209) gene set was positively enriched after HGP administration (Figure 5H). Specifically, compared with the control group, the model group exhibited significantly downregulated expression of antioxidant-related genes such as *Nqo1*, *Prdx1*, *Prxl2b*, and *Srxn1*, as well as significantly downregulated expression of glutathione metabolism enzyme-related genes including *Mgst3*, *Gstt2*, *Gsta2*, *Gsta1*, *Gpx4*, and *Gsr*. In contrast, the expression of hemoglobin α- and β-chain-related genes such as *Hbb-bs*, *Hba-a2*, *Hba-a1*, and *Hbb-bt* was significantly upregulated. These genes showed counter-directional expression changes following HGP treatment (Figure 5K). Collectively, the transcriptomic results indicate that HGP treatment was associated with coordinated expression changes in genes related to glutathione metabolism, PPAR signaling, and antioxidant responses. These findings provide exploratory transcriptional evidence for the involvement of these biological processes in the effects of HGP against ALI.

### 2.5. Integrated Transcriptomic and Metabolomic Analysis Identifies Biological Processes Associated with HGP Intervention in ALI

**Figure 6:**
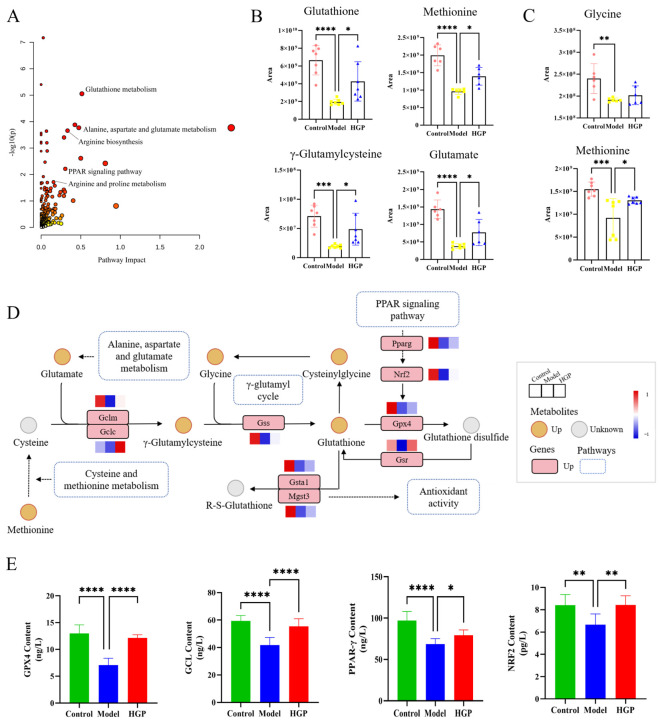
Integrated transcriptomic and metabolomic analysis results. (**A**): Integrated metabolic pathway map; (**B**): Bar plot of peak areas of key metabolites in liver tissue (*n* = 7 per group; compared with the model group, * *p* < 0.05, *** *p* < 0.001, **** *p* < 0.0001); (**C**): Bar plot of peak areas of key metabolites in serum (*n* = 7 per group; compared with the model group, * *p* < 0.05, ** *p* < 0.01,*** *p* < 0.001); (**D**): Metabolic network diagram of key genes and metabolites, the arrows represent the direction of the metabolic flow.; (**E**): Concentrations of selected proteins in liver homogenate supernatants of mice in each group; Data are presented as mean ± SD and were analyzed by one-way ANOVA followed by Tukey’s multiple-comparison test. (*n* = 6 per group; compared with the model group, * *p* < 0.05, *** *p* < 0.001, **** *p* < 0.0001).

Integrated hepatic transcriptomic and metabolomic analysis was performed using the 181 DEGs that met the stringent criteria in both comparisons and showed opposite expression directions, together with the selected putatively annotated liver metabolites showing counter-directional trends following HGP treatment. Joint pathway analysis identified 38 pathways with *p* < 0.05 (Figure 6A). Pathways containing both gene and metabolite inputs and supported by the individual omics findings were further prioritized for visualization. These pathways mainly involved alanine, aspartate, and glutamate metabolism; glutathione metabolism; the γ-glutamyl cycle; cysteine and methionine metabolism; and PPAR signaling (Figure 6D). Bar plots of peak areas for key metabolites involved in these processes are shown in Figure 6B,C. As shown in Figure 5I–K, the selected genes associated with glutathione metabolism, PPAR signaling, and antioxidant activity showed decreased expression in the model group and counter-directional changes following HGP treatment. Consistently, the concentrations of selected proteins involved in the proposed pathway were measured by ELISA. Compared with the control group, the hepatic protein concentrations of GPX4, glutamate-cysteine ligase (GCL), PPARγ, and NRF2 in the liver tissue of model group mice were significantly decreased (Figure 6E). Compared with the model group, the hepatic levels of these key proteins in the HGP-treated group were significantly increased. These findings demonstrate directional concordance between the selected transcriptomic and protein-level measurements. These findings suggest that the effects of HGP may be associated with glutathione metabolism and a PPARγ/NRF2/GPX4-related antioxidant network.

### 2.6. Molecular Docking Analysis Predicts Interactions Between Key Components of HGP and Targets

Our team previously conducted a comprehensive analysis of the chemical constituents of HGP. Based on the principles of specificity, measurability, efficacy, and formulation compatibility for quality markers, the chemical components of HGP were systematically screened. 9 compounds were preliminarily identified as potential quality markers of HGP: saikosaponin b2, chlorogenic acid, (*R*,*S*)-goitrin, schisandrin A, schisandrin, schisantherin A, schisandrol B, schisandrin C, and glycohyodeoxycholic acid [16]. Therefore, molecular docking experiments were performed between the nine potential quality markers of HGP and the key pathway proteins GPX4, GCL, PPARγ, and NRF2. The binding energies between the 9 potential quality markers and the proteins are shown in Table S9. The negative binding energies suggested possible binding potential between selected HGP quality markers and the selected proteins. These results were considered exploratory computational evidence and were not interpreted as direct proof of target engagement. GPX4, GCL, PPARγ, and NRF2 were selected as representative proteins for exploratory docking analysis of these nine quality markers. Because GCL is composed of catalytic and modifier subunits, GCLC and GCLM were used for docking analysis. Representative docking conformations are shown in Figures S5–S7. Predicted binding energies below −5.0 kcal/mol suggested favorable potential interactions between selected HGP quality markers and the examined proteins. These results suggested favorable potential interactions between selected HGP quality markers and the examined proteins.

## 3. Discussion

ALT and AST are commonly used indicators of hepatocellular injury, whereas CDT primarily reflects chronic alcohol exposure [24,25]. To some extent, these parameters reflect the extent of alcohol-induced hepatocyte damage and dysfunction. Experimental data showed that HGP reduced serum ALT, AST, and CDT levels in ALI mice indicate that HGP treatment was associated with reduced biochemical evidence of hepatocellular injury. Chronic alcohol consumption leads to abnormal hepatic lipid deposition, resulting in steatosis, which may progress to steatohepatitis. The experimental results demonstrated that HGP effectively suppressed the abnormal elevation of hepatic TG levels in ALI mice. GSH is one of the most important intracellular antioxidants, while ROS serve as key markers of oxidative stress. During alcohol metabolism, ethanol generates excessive ROS via pathways such as CYP2E1, disrupting the intracellular redox balance, manifested by GSH depletion and ROS accumulation, thereby inducing oxidative damage in hepatocytes [6]. Experimental results showed that HGP increased GSH levels and reduced ROS levels in the liver of ALI mice. This suggests that HGP treatment was associated with increased hepatic GSH levels and reduced ROS accumulation. Alternatively, these findings may involve NRF2-related antioxidant responses, upregulate the expression of key antioxidant enzymes such as GPXs, and thereby enhance the capacity of cells to scavenge free radicals [26,27]. TNF-α and IL-1β are key pro-inflammatory cytokines that play important roles in the progression of ALI by mediating inflammatory responses. Alcohol metabolites, such as acetaldehyde, activate Kupffer cells and neutrophils, leading to substantial release of TNF-α and IL-1β, which in turn trigger inflammatory reactions and exacerbate liver injury. The reductions in hepatic TNF-α and IL-1β levels indicate an attenuation of the inflammatory response following HGP treatment [28]. Among the tested doses, the high-dose group showed relatively larger changes in several measured endpoints. Previous work from our group also evaluated HGP in a choline-deficient, ethionine-supplemented mouse model of NASH-related hepatic fibrosis. HGP attenuated hepatic fibrosis and was associated with alterations in gut microbiota and bile acid metabolism [29]. These findings suggest that the hepatoprotective effects of HGP may also extend to metabolically driven liver injury, although alcohol-induced liver injury and MASLD differ in their underlying pathogenesis.

Numerous studies have shown that the pathological mechanisms of ALI are closely associated with oxidative stress, inflammation, lipid metabolism disorders, and fibrosis [30,31]. Network pharmacology studies have shown that HGP can modulate the expression of biological processes and molecular functions related to oxidative stress and inflammation. Among these, glutathione metabolism dysfunction is closely associated with the pathological mechanisms of ALI, where impaired synthesis, increased consumption, and impaired regeneration form a vicious cycle that further exacerbates liver injury. On one hand, this leads to an oxidative stress cascade: decreased GSH levels result in reduced ROS scavenging capacity, triggering lipid peroxidation (e.g., elevated malondialdehyde levels), oxidative damage to mitochondrial DNA (e.g., accumulation of 8-hydroxy-2′-deoxyguanosine), and inactivation of protein functions [32]. On the other hand, this exacerbates inflammation and fibrosis. Oxidative stress activates TGF-β1/NF-κB/COX II pathway, amplifying the inflammatory response [33]. GSH deficiency enhances TGF-β signaling in hepatic stellate cells, promoting collagen deposition and fibrosis [34]. Additionally, insufficient GSH levels upregulate sterol regulatory element-binding protein 1c (SREBP-1c) activity, increase fatty acid synthase expression, and exacerbate hepatic steatosis, leading to lipid metabolism disorders [35]. Therefore, glutathione metabolism may represent a key pathway through which HGP intervenes in ALI.

Non-targeted metabolomics analysis of liver and serum revealed that HGP modulated multiple metabolic pathways in ALI mice. Among the shared pathways in both serum and liver tissues, glutathione metabolism; alanine, aspartate, and glutamate metabolism; arginine and proline metabolism; purine metabolism; nicotinate and nicotinamide metabolism; and glyoxylate and dicarboxylate metabolism all contribute to maintaining glutathione synthesis and function by providing precursors, regulating redox status, or participating in detoxification processes. However, metabolite-level analysis alone is insufficient to fully explain the complexity of signal transduction underlying HGP intervention in ALI. Therefore, transcriptomic and metabolomic data were integrated to analyze the mechanism of HGP action at both the gene and metabolic levels. Transcriptomic analysis identified DEGs between groups, and revealed that HGP treatment was associated with transcriptional changes opposing a subset of alcohol-induced alterations. GO, KEGG, and GSEA results indicated that HGP may exert its effects by regulating core genes involved in glutathione metabolism and PPAR signaling pathway, suggesting the involvement of antioxidant-related transcriptional responses. The glutathione metabolism pathway primarily encompasses glutathione synthesis, the glutathione redox cycle, and glutathione degradation, and mainly occurs in the liver. Glutathione plays a critical role in counteracting oxidative stress, participating in the detoxification of xenobiotics, determining cellular redox status, and regulating processes such as cell growth and apoptosis. Dysregulation of this pathway is directly associated with liver disease, further underscoring its importance in liver health and disease. Recent studies have highlighted that glutathione metabolism plays a significant role in the pathological progression of ALI [7,36]. A combined analysis based on transcriptomic data and predicted metabolic flux in liver tissues from patients with ALI revealed that glutathione metabolism and transport activity become progressively dysregulated with increasing severity of ALI [37]. The PPAR signaling pathway is mainly composed of three subtypes: PPARα, PPARβ/δ, and PPARγ. Among these, PPARγ exerts multiple physiological functions, including antioxidant stress and inflammatory regulation [38,39]. PPARγ can form a heterodimer with the retinoid X receptor (RXR) and bind to the PPAR response element (PPRE) in the promoter regions of target genes, thereby participating in the regulation of genes involved in antioxidant stress responses. Upon activation, the PPARγ-RXR heterodimer specifically recognizes and binds to the PPRE in DNA, subsequently modulating the transcriptional levels of downstream target genes. Current studies have shown that activation of the NRF2-GPX4 axis can prevent and ameliorate ethanol-induced hepatocyte injury [40]. Studies have suggested potential interactions between the PPARγ and NRF2-related pathways. The promoter region of the *Nfe2l2* gene contains a PPAR response element, and PPARγ may influence *Nfe2l2* transcription under specific experimental conditions [12]. Therefore, coordinated changes in PPARγ, NRF2 and GPX4 may contribute to the antioxidant response involved in ALI. In this study, ELISA was used to validate hepatic GPX4, GCL, PPARγ, and NRF2 levels. The results showed that HGP treatment increased the measured concentrations of these proteins. These coordinated changes may be associated with the hepatoprotective effects of HGP. The coordinated changes in PPARγ, NRF2, GPX4, and glutathione metabolism-related indicators suggest the involvement of a PPARγ/NRF2/GPX4-related antioxidant network.

Molecular docking was used as an exploratory computational analysis to evaluate the potential interactions between representative HGP quality markers and selected proteins involved in the proposed pathway. The results provided preliminary supportive information but should not be regarded as direct evidence of target engagement. Several limitations should be acknowledged. Only male mice were used, and daily liquid-diet intake was not quantitatively recorded, limiting the evaluation of sex-related responses and potential differences in energy intake. Histopathological findings were assessed qualitatively because semiquantitative scoring and Oil Red O-positive area quantification were not performed. The multi-omics analyses included only the HGP-H group; therefore, the findings cannot establish dose-dependent molecular effects, be directly extrapolated to the lower-dose groups, or support adjustment of the recommended clinical dose. In addition, the network pharmacology analysis was based primarily on human target annotations and was conducted independently of the mouse omics analyses without human-to-mouse ortholog conversion, which limits direct cross-species interpretation. RNA-seq was performed with three biological replicates per group without independent qRT-PCR validation, and the metabolites were putatively annotated at MSI Level 2 without authentic-standard confirmation or targeted quantification. The absence of a prospectively defined outlier-handling criterion may have introduced potential analytical bias. Finally, functional or genetic intervention experiments were not performed. Thus, the proposed PPARγ/NRF2/GPX4-related antioxidant network should be interpreted as an associative mechanism that requires further validation.

## 4. Materials and Methods

### 4.1. Chemicals and Reagents

Lieber-DeCarli control and ethanol liquid diet were purchased from Beijing SPF Biotechnology Co., Ltd. (Beijing, China). HuGan Tablets (HGP) are a commercially available traditional Chinese medicine compound tablet manufactured by Heilongjiang Sunflower Pharmaceutical Co., Ltd. (Harbin, China; approval number: Z20003336; batch number: 202203019). HGP was used as the commercial tablet preparation rather than a laboratory-prepared extract. Before administration, the tablets were ground into powder and freshly suspended in distilled water to the required concentrations for intragastric administration. The chemical constituents and potential quality markers of HGP were characterized in our previous LC–MS-based studies [16,17]. LC-MS-grade ammonium formate was purchased from Honeywell (Offenbach, Germany). LC–MS-grade methanol, acetonitrile, and formic acid were purchased from Thermo Fisher Scientific (Dover, DE, USA). 4% paraformaldehyde fixative was purchased from Wuhan Servicebio Technology Co., Ltd. (Wuhan, China). Unless otherwise stated, all other reagents were of analytical grade.

### 4.2. Establishment, Grouping, and Dosing Regimen of the ALI Model

All animal experiments and procedures were approved by the Experimental Animal Ethics Committee of Beijing University of Chinese Medicine (Approval No. BUCM-2024031303-1151) and were conducted in strict accordance with the Regulations for the Administration of Laboratory Animals and the Guidelines for the Use and Care of Vertebrate Animals.

SPF male C57BL/6J mice, aged 6 weeks and weighing 20 ± 2 g, were purchased from Beijing SPF Biotechnology Co., Ltd. (Beijing, China). All mice were housed in the Animal Experimental Center of Beijing University of Chinese Medicine under controlled conditions: relative humidity of 55–65%, temperature of 21–25 °C, and a 12 h light/dark cycle. Prior to the experiment, the mice had ad libitum access to food and water and were acclimated for 7 days, including 4 days of environmental adaptation with solid diet and free access to water, followed by 3 days of adaptation to the Lieber-DeCarli control liquid diet.

Male mice were selected to maintain consistency with commonly used Lieber–DeCarli model protocols and to reduce additional variability associated with sex-dependent differences in ethanol metabolism and metabolic responses. After the adaptation period, the mice were assigned to six groups using a random-number table (n = 8 per group): (1) control group; (2) model group (Lieber-DeCarli ethanol liquid diet); (3) silibinin group (silibinin, 0.3452 g·kg^−1^·day^−1^); (4) high-dose HGP group (HGP-H, 3.4524 g·kg^−1^·day^−1^); (5) medium-dose HGP group (HGP-M, 1.7262 g·kg^−1^·day^−1^); (6) low-dose HGP group (HGP-L, 0.8631 g·kg^−1^·day^−1^). The control group received the Lieber-DeCarli control liquid diet. The other groups received the Lieber-DeCarli ethanol liquid diet at concentrations of 1%, 2%, 3%, 4%, and 5% (each for one day) as an alcohol adaptation period. Subsequently, the control group continued to receive the control liquid diet, while groups (2)–(6) were fed a 5% Lieber-DeCarli ethanol liquid diet for 8 weeks. From week 5 onward, the control and model groups received normal saline by gavage, while groups (3)–(6) received silibinin or different doses of HGP by gavage for 4 weeks (weeks 5–8). The phenotype of the ALI model was evaluated based on the combined changes in serum ALT and AST levels, hepatic TG accumulation, liver index, and histopathological alterations.

The low dose of HGP was set as the mouse equivalent dose converted from the clinically recommended dose of the commercial tablet preparation based on the body surface area normalization method. The medium and high doses were set at two- and four-fold of the low dose, respectively, to evaluate the dose–response relationship. Therefore, the doses of HGP-L, HGP-M, and HGP-H were 0.8631, 1.7262, and 3.4524 g·kg^−1^·day^−1^, respectively. Body weight was recorded every two days, and the liquid diet was replaced daily.

After the final administration, serum and liver samples were collected. The liver index for each mouse was calculated based on body weight before dissection and liver wet weight, using the following formula: liver index (mg/g) = liver wet weight (mg)/body weight (g).

### 4.3. Biochemical Assays

Serum ALT and AST levels and hepatic TG and GSH levels were determined using commercial assay kits purchased from Nanjing Jiancheng Bioengineering Institute (Nanjing, China) according to the manufacturers’ instructions. For liver biochemical measurements, liver tissues were homogenized for 120 s and centrifuged, and the supernatants were collected for subsequent analysis. Serum CDT and hepatic TNF-α, IL-1β, and ROS levels were measured using commercial ELISA kits obtained from Jiangsu Kete Biotechnology Co., Ltd. (Yancheng China) according to the manufacturers’ protocols. Serum and liver samples were identified using coded labels during biochemical measurements, and the investigators conducting the assays were unaware of the corresponding group allocation.

### 4.4. Histopathological Examination

For hematoxylin and eosin staining, liver tissues were fixed in 4% paraformaldehyde, embedded in paraffin, sectioned, deparaffinized, and stained with hematoxylin and eosin. Histopathological changes, including steatosis, hepatocellular injury, and inflammatory cell infiltration, were qualitatively examined under a light microscope, and representative fields were photographed.

For Oil Red O staining, fresh liver tissues were embedded in OCT compound and cryosectioned. The sections were fixed and stained with Oil Red O working solution, followed by hematoxylin counterstaining. Hepatic lipid accumulation was qualitatively evaluated under a light microscope, and representative fields were photographed.

Histological sections were assigned coded labels before examination, and the investigators conducting the qualitative assessment were unaware of the corresponding group allocation. For each animal, 6 non-overlapping fields per section were observed under the same magnification. Histopathological changes and hepatic lipid accumulation were evaluated qualitatively.

### 4.5. Network Pharmacology Target Screening and Pathway Enrichment

Using “Alcoholic Liver Disease,” “Alcoholic Hepatitis,” and “Alcoholic Fatty Liver” as keywords, disease-related targets were retrieved from the OMIM (https://omim.org/, accessed on 3 January 2024) and GeneCards (https://www.genecards.org/, accessed on 3 January 2024) databases. These broader alcohol-related liver disease terms were used to improve the coverage of pathological processes relevant to experimental ALI. After merging and removing duplicates, the alcohol-related liver disease-associated target set was obtained. Our previous study identified 128 compounds and 29 in vivo metabolites from HGP [16]. The targets of these 157 compounds were retrieved from the Swiss Target Prediction (https://swisstargetprediction.ch/, accessed on 3 June 2024) and ETCM (http://www.tcmip.cn/ETCM/, accessed on 3 June 2024) databases and defined as HGP-related targets. No additional database score threshold was applied, and all retrieved targets were retained after removal of duplicate entries. Venn diagrams were used to identify the intersection between HGP-related targets and ALD-related targets, representing predicted candidate targets potentially relevant to HGP intervention in ALI. Common targets were imported into the STRING platform with the species set to Homo sapiens, and the PPI network was visualized using Cytoscape v3.10.3 (https://cytoscape.org/, accessed on 15 June 2024). Topological analysis was performed using the CytoNCA plugin, and targets with degree values greater than the median were identified as core predicted candidate targets for HGP intervention in ALI.

These core predicted candidate targets were submitted to the DAVID database for GO and KEGG enrichment analysis, with the species set to Homo sapiens. GO analysis included BP, CC, and MF. Enrichment results were visualized using the Bioinformatics Platform (https://www.bioinformatics.com.cn/, accessed on 17 June 2024).

### 4.6. Non-Targeted Metabolomics Analysis

#### 4.6.1. Sample Preparation

Based on the overall pharmacodynamic findings, the HGP-H group showed relatively larger improvements in several measured endpoints and was therefore selected for subsequent exploratory multi-omics and validation analyses. For liver and serum non-targeted metabolomics analyses, seven biological replicates from each of the control, model, and HGP-H groups were included. Samples were randomly selected for omics analysis.

Liver sample preparation: Liver tissues were thawed on ice and placed in 2 mL centrifuge tubes. Twenty volumes of pre-cooled 80% methanol were added, and the samples were mechanically homogenized until no visible tissue clumps remained. The homogenates were incubated at −20 °C for 1 h to precipitate proteins. After incubation, samples were centrifuged at 13,000 rpm for 15 min at 4 °C. Aliquots of 120 μL of supernatant were transferred to new centrifuge tubes and dried under nitrogen gas at room temperature. The residues were reconstituted with 50 μL of 80% methanol, vortexed for 1 min, and centrifuged again at 13,000 rpm for 10 min at 4 °C. The supernatants were transferred to vials with inserts for analysis.

Serum sample preparation: For each mouse, 50 μL of serum was placed in a 2 mL centrifuge tube. Four volumes of pre-cooled methanol were added, vortexed for 2 min, and incubated at −20 °C for 1 h to precipitate proteins. After incubation, samples were centrifuged at 13,000 rpm for 15 min at 4 °C. Aliquots of 160 μL of supernatant were transferred to new centrifuge tubes and dried under nitrogen gas at room temperature. The residues were reconstituted with 80 μL of 80% methanol, vortexed for 1 min, and centrifuged at 13,000 rpm for 10 min at 4 °C. The supernatants were transferred to vials with inserts for analysis. QC samples were prepared by mixing 60 μL from each sample and processed identically.

#### 4.6.2. Chromatographic and Mass Spectrometric Conditions

Chromatographic separation was performed using a Thermo Scientific Vanquish UHPLC system equipped with an ACQUITY UPLC BEH C18 column (2.1 mm × 100 mm, 1.7 μm, Dover, DE, USA). The injection volume was 1 μL. The mobile phase consisted of 0.1% formic acid in water (A) and acetonitrile (B) with the following gradient elution: 0–0.3 min, 2–5% B; 0.3–1 min, 5–20% B; 1–3 min, 20–40% B; 3–4.5 min, 40–60% B; 4.5–6 min, 60–80% B; 6–11 min, 80–90% B; 11–11.5 min, 90–95% B; 11.5–13.5 min, 95–5% B; 13.5–14 min, 5–2% B; 14–15 min, 2% B. The flow rate was 0.2 mL/min. Mass spectrometry was performed using a Thermo Q Exactive Plus mass spectrometer equipped with an electrospray ionization (ESI) source (Dover, DE, USA). The spray voltages were set to 3.8 kV (positive ion mode) and 3.2 kV (negative ion mode). Data-dependent acquisition (DDA) mode was used to collect data in both positive and negative ion modes. The resolutions for MS and MS/MS were 70,000 and 17,500, respectively, with a scan range of *m/z* 70–1050.

#### 4.6.3. Quality Control and Analytical Stability Assessment

Quality control was assessed in terms of instrument precision, sample-preparation repeatability, and analytical-sequence stability. Pooled quality control (QC) samples were prepared as described in Section 4.6.1 and processed using the same procedure as the study samples.

To evaluate sample-preparation repeatability, six QC replicates were prepared in parallel and analyzed. Instrument precision was assessed by six consecutive injections of the same QC sample after the analytical system had stabilized. During the analytical sequence, one QC sample was injected after every six study samples to monitor system stability. Representative ions covering different retention times and signal intensities were selected from the total ion chromatograms. The relative standard deviations of retention times and peak areas were calculated to evaluate the reproducibility and stability of the analytical method. Periodic QC injections were used to monitor analytical stability and potential signal drift throughout the analytical sequence. All study samples were analyzed within the same analytical sequence. The relative standard deviations of the peak areas of representative ions were calculated to evaluate analytical reproducibility and stability. An RSD of less than 20% was used as the acceptance criterion, and all calculated RSD values met this criterion.

#### 4.6.4. Data Processing

Raw data were converted using ABF Converter v2.3.7 and processed using MS-DIAL for peak extraction, alignment, matching, and intensity correction. Isotopic peaks were removed, and the absolute peak intensity threshold was set to 200. The 80% rule was applied to remove compounds with missing values. The resulting data matrix was imported into SIMCA 14.1 for multivariate statistical analysis. PCA was used to evaluate the overall metabolic profiles of the samples. OPLS-DA was subsequently performed to assess group discrimination. The goodness-of-fit and predictive performance of the OPLS-DA models were evaluated using R^2^Y and Q^2^ values, respectively. Model predictive performance was assessed by cross-validation, and the risk of overfitting was further evaluated using 200 permutation tests. Multiple-testing correction was performed using the Benjamini–Hochberg method.

Candidate differential metabolites between the control and model groups were screened based on adjusted *p* value < 0.05 and VIP > 1. Metabolite annotation was based on accurate precursor masses, retention-time alignment, MS/MS fragment ions, and database matching against HMDB, MassBank, METLIN, and mzCloud. The mass tolerances for both precursor and fragment ions were set to 5 ppm, and the matching results were further manually inspected. Because no authentic standards were used to confirm retention times and MS/MS spectra, the metabolites were classified as putatively annotated compounds at Metabolomics Standards Initiative Level 2. Metabolic pathway enrichment analysis was performed using MetaboAnalyst 6.0 (https://www.metaboanalyst.ca/, accessed on 8 July 2024).

### 4.7. Transcriptomics Analysis

Three independent liver samples from each of the control, model, and HGP-H groups were used for transcriptomic analysis, and no samples were pooled. RNA extraction, library construction, and sequencing were performed by Majorbio Bio-Pharm Technology Co., Ltd. (Shanghai, China). Libraries were prepared using the Illumina Stranded mRNA Prep, Ligation kit (Baltimore, MD, USA) and sequenced on an Illumina NovaSeq X Plus platform with paired-end 150 bp reads. Raw sequencing reads were filtered to obtain clean reads and aligned to the *Mus musculus* reference genome (GRCm39). Transcripts Per Million reads (TPM) values were used for expression visualization. Gene expression levels were quantified using RSEM v1.3.3, and differential-expression analysis was performed using the DESeq2 package in R v4.5.2 [41]. RNA quality, sequencing quality, and mapping statistics were evaluated before downstream analysis.

Genes with a nominal *p* value < 0.05 were initially summarized to provide a descriptive overview of transcriptional changes and were visualized in supplementary volcano plots. For subsequent intersection and functional enrichment analyses, differentially expressed genes (DEGs) were defined as those with an adjusted *p* value < 0.05 and |log2FoldChange| > 0.585. Multiple-testing correction was performed using the Benjamini–Hochberg method. Genes meeting these criteria in both the Model versus Control and HGP versus Model comparisons and exhibiting opposite directions of change were retained for further analysis.

GO and KEGG enrichment analyses were performed using GOATOOLS v2.3 and the Python v3.10 SciPy package v1.12.0, respectively. Gene set enrichment analysis (GSEA) was conducted on the Majorbio Cloud Platform using the complete ranked gene list from the HGP versus Model comparison, with gene sets obtained from the Molecular Signatures Database (MSigDB). For gene-level visualization, genes with a nominal *p* value < 0.05 in both the Model versus Control and HGP versus Model comparisons were screened. Genes showing opposite expression directions between the two comparisons and belonging to the glutathione metabolism, PPAR signaling, or antioxidant activity gene sets were selected for display in Figure 5I–K.

### 4.8. Integrated Transcriptomic and Metabolomic Analysis

For integrated transcriptomic and metabolomic analysis, DEGs meeting an adjusted *p* value < 0.05 and |log2FoldChange| > 0.585 in both the Model versus Control and HGP versus Model comparisons and showing opposite directions between the two comparisons were included. Liver metabolites meeting VIP > 1 and an adjusted *p* value < 0.05 in the Control versus Model comparison and showing counter-directional changes following HGP treatment were also included. The selected genes and metabolites were submitted to MetaboAnalyst 6.0 for joint pathway analysis, and pathways with *p* < 0.05 were retained.

The pathways highlighted in Figure 6D were selected from the significantly enriched pathways based on their inclusion of the selected gene or metabolite nodes, their consistency with the independent transcriptomic and metabolomic findings, and their relevance to glutathione metabolism, amino acid metabolism, and PPAR-related responses. The displayed nodes were genes and metabolites mapped to these selected pathways and showing counter-directional changes following HGP treatment.

### 4.9. Validation of Key Proteins Expression in Identified Pathways

For ELISA-based validation, six liver samples from each of the control, model, and HGP-H groups were used. Approximately 100 mg of liver tissue was homogenized in 900 μL of ice-cold homogenization buffer at a tissue-to-buffer ratio of 1:9 (*w*/*v*). The homogenates were centrifuged, and the supernatants were collected for analysis. The concentrations of GPX4, GCL, PPARγ, and NRF2 in the liver homogenate supernatants were measured using commercial ELISA kits obtained from Jiangsu Kete Biotechnology Co., Ltd. (Yancheng, China) according to the manufacturer’s instructions. The results were reported as the measured concentrations in the homogenate supernatants. Total protein concentrations were not measured; therefore, the ELISA results were not normalized to total protein content.

### 4.10. Molecular Docking of HGP Active Components and Key Pathway Proteins

The 3D structures of potential quality markers of HGP were obtained from the PubChem database: saikosaponin b2 (PubChem CID: 21637642), chlorogenic acid (PubChem CID: 1794427), (R,S)-goitrin (PubChem CID: 3034683), schisandrin (PubChem CID: 11102092), schisandrol B (PubChem CID: 634470), schisantherin A (PubChem CID: 151529), schisandrin A (PubChem CID: 155256), schisandrin C (PubChem CID: 119112), and glycohyodeoxycholic acid (PubChem CID: 114611). The 3D structures of key pathway proteins were obtained from the Protein Data Bank (PDB) and UniProt databases: GPX4 (PDB ID: 6elw), GCLM (UniProt: P48507), GCLC (UniProt: P48506), PPARγ (PDB ID: 9f7w), and NRF2 (PDB ID: 5wfv). Molecular docking was performed using AutoDock Vina 1.2.2. After generating binding conformations, PyMOL v3.0 was used to visualize the binding modes between ligands and receptors and to analyze key interactions such as hydrogen bonds and hydrophobic interactions. Predicted binding energies below −5.0 kcal/mol were considered to indicate favorable potential ligand–protein interactions. More negative binding-energy values were interpreted as suggesting more favorable predicted binding.

### 4.11. Statistical Analysis

Data were analyzed using SPSS 20.0 and visualized using GraphPad Prism 8. Results are presented as the mean ± standard deviation. Normality was assessed using the Shapiro–Wilk test, and homogeneity of variance was evaluated using Levene’s test. For normally distributed data with homogeneous variances, comparisons among multiple groups were performed using one-way analysis of variance followed by Tukey’s multiple-comparison test. For data that did not meet these assumptions, the Kruskal–Wallis test followed by Dunn’s multiple-comparison test was used. A two-sided *p* value < 0.05 was considered statistically significant. During the original analysis, two animals from each group were excluded from the pharmacodynamic analyses based on inspection of extreme values rather than a prospectively defined statistical criterion, resulting in a final sample size of six animals per group.

## 5. Conclusions

HGP alleviated alcohol-induced liver injury in mice, as indicated by improvements in liver injury-related biochemical parameters, hepatic lipid accumulation, oxidative stress, inflammatory responses, and histopathological alterations. Metabolomic and transcriptomic analyses identified changes associated with hepatic glutathione metabolism, PPAR signaling, and antioxidant-related biological processes. Consistent with these findings, ELISA measurements showed increased concentrations of GPX4, GCL, PPARγ, and NRF2 in liver homogenate supernatants following HGP treatment. Together, these experimental findings suggest that the hepatoprotective effects of HGP may be associated with changes in hepatic glutathione metabolism and a PPARγ/NRF2/GPX4-related antioxidant network. However, the present results do not establish a causal regulatory relationship among these proteins. Network pharmacology and molecular docking provided only exploratory, hypothesis-generating information and should not be interpreted as evidence of direct target engagement or pathway activation. Further studies using targeted metabolite quantification, independent gene-expression validation, and functional intervention experiments are required to confirm the proposed mechanisms.

## Data Availability

The original contributions presented in the study are included in the article and Supplementary Materials. Further inquiries can be directed to the corresponding author. The raw RNA-seq data have been submitted to the NCBI Sequence Read Archive under the temporary submission ID SUB16141457. The public accession number will be added to the manuscript once it has been assigned. The raw metabolomics data are available from the corresponding author upon reasonable request.

## Supplementary Materials

The following supporting information can be downloaded at: https://www.mdpi.com/article/10.3390/ph19071007/s1, Figure S1: Body weight and liver wet weight of mice in each experimental group; Figure S2: Quality assessment of transcriptomic sequencing data; Figure S3: Distribution of gene expression levels among transcriptomic samples; Figure S4: Overview and functional enrichment analysis of hepatic transcriptomic changes; Figure S5: Predicted docking conformations between selected HGP quality markers and PPARγ/GPX4; Figure S6: Predicted docking conformations between selected HGP quality markers and GCLC/GCLM; Figure S7: Predicted docking conformations between selected HGP quality markers and NRF2; Table S1: Putatively annotated candidate metabolites showing counter-directional trends following HGP-H treatment in liver under positive ion mode; Table S2: Putatively annotated candidate metabolites showing counter-directional trends following HGP-H treatment in liver under negative ion mode; Table S3: Putatively annotated candidate metabolites showing counter-directional trends following HGP-H treatment in serum under positive ion mode; Table S4: Putatively annotated candidate metabolites showing counter-directional trends following HGP-H treatment in serum under negative ion mode; Table S5: RNA quality assessment results; Table S6: Sequencing data quality-control results; Table S7: Alignment statistics of clean reads against the reference genome; Table S8: DEGs meeting the stringent criteria in both comparisons and showing opposite expression directions following HGP treatment; Table S9: Predicted binding energies between nine potential HGP quality markers and selected proteins.
