# Supplementary material for: Hugan Tablets Alleviate Alcoholic Liver Injury by Modulating Hepatic Glutathione Metabolism and PPARγ/NRF2/GPX4-Related Antioxidant Defense
Source: Pharmaceuticals (Basel). 2026 Jun 29;19(7):1007. doi: 10.3390/ph19071007 (PMC13414623; doi:10.3390/ph19071007)
Supplement: Supplementary file 1 [file pharmaceuticals-19-01007-s001.zip › pharmaceuticals-4343244-supplementary.pdf]

Supplementary Material

Supplementary Figures

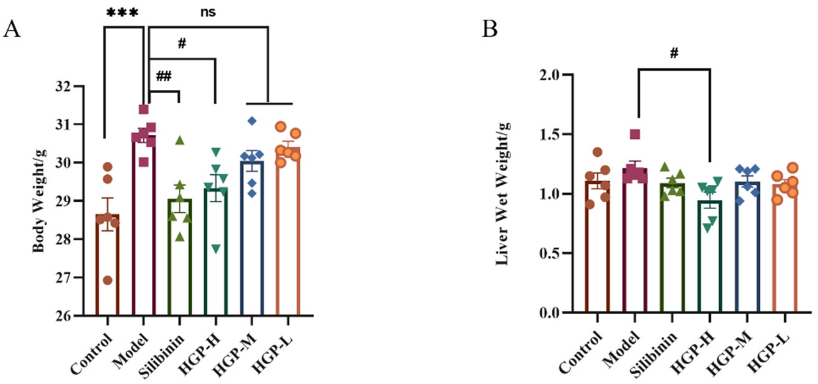

Figure S1. Final body weight and liver wet weight of mice after 8 weeks. (A) Final body weight. (B) Liver wet weight. Data are presented as mean ± SD (n = 6) and analyzed by one-way ANOVA followed by Tukey's multiple-comparison test. $^{*}P < 0.05$, $^{**}P < 0.01$, $^{***}P < 0.001$ versus the Control group; $^{\#}P < 0.05$, $^{\#\#}P < 0.01$, $^{\#\#\#}P < 0.001$ versus the Model group.

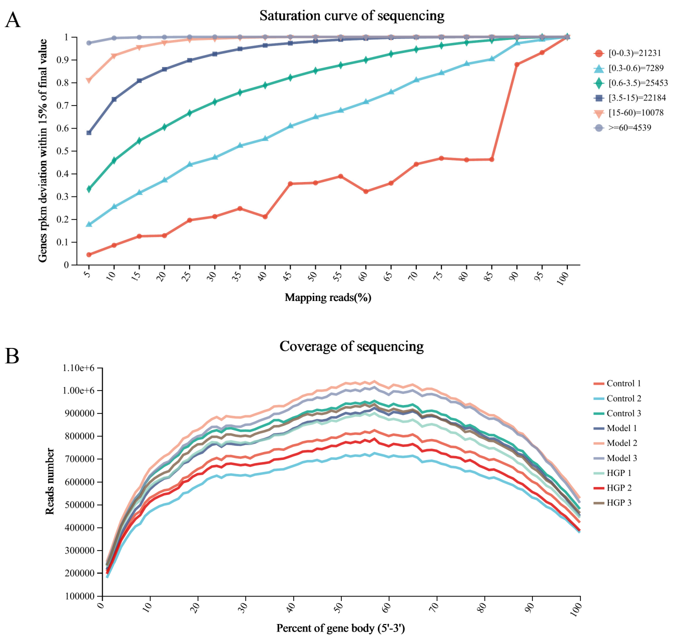

Figure S2. Quality assessment of transcriptome sequencing alignment results. A: sequencing saturation analysis; **B**: Gene-body coverage analysis.

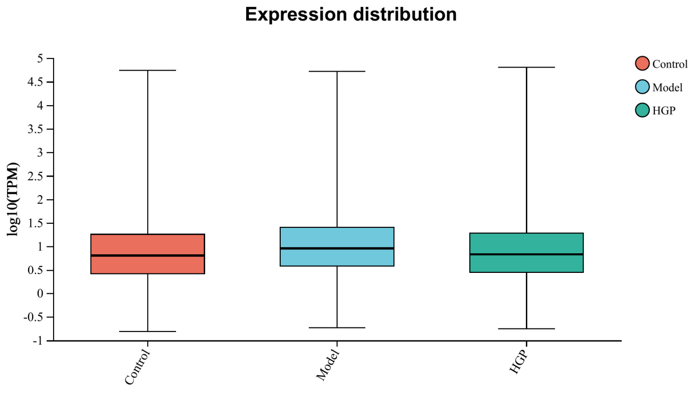

Figure S3. Box plot of gene expression levels.

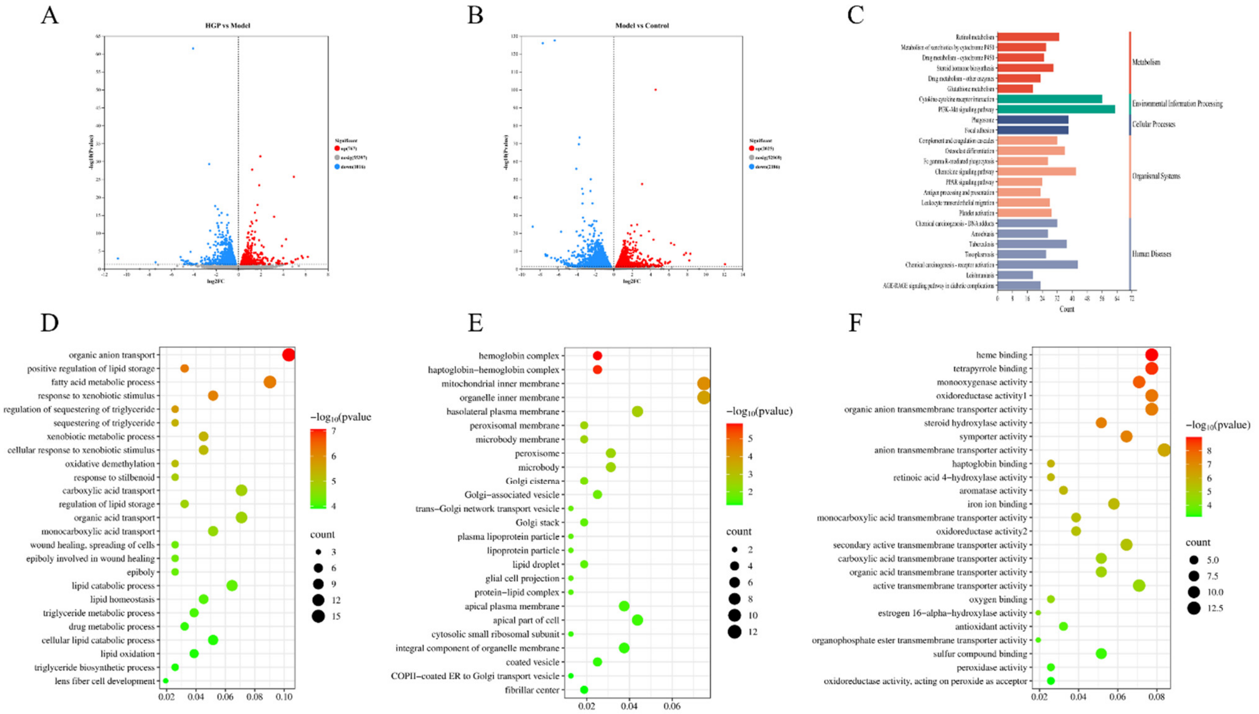

Figure S4. Overview and functional enrichment analysis of hepatic transcriptomic changes. A: Volcano plot of genes with a $P < 0.05$ between the control and model groups; **B**: Volcano plot of genes with a $P < 0.05$ between the model and HGP-H groups; **C**: KEGG enrichment analysis of DEGs between the control and model groups; **D**: GO BP enrichment analysis of DEGs between the control and model groups; **E**: GO CC enrichment analysis of DEGs between the control and model groups; **F**: GO MF enrichment analysis of DEGs between the control and model groups.

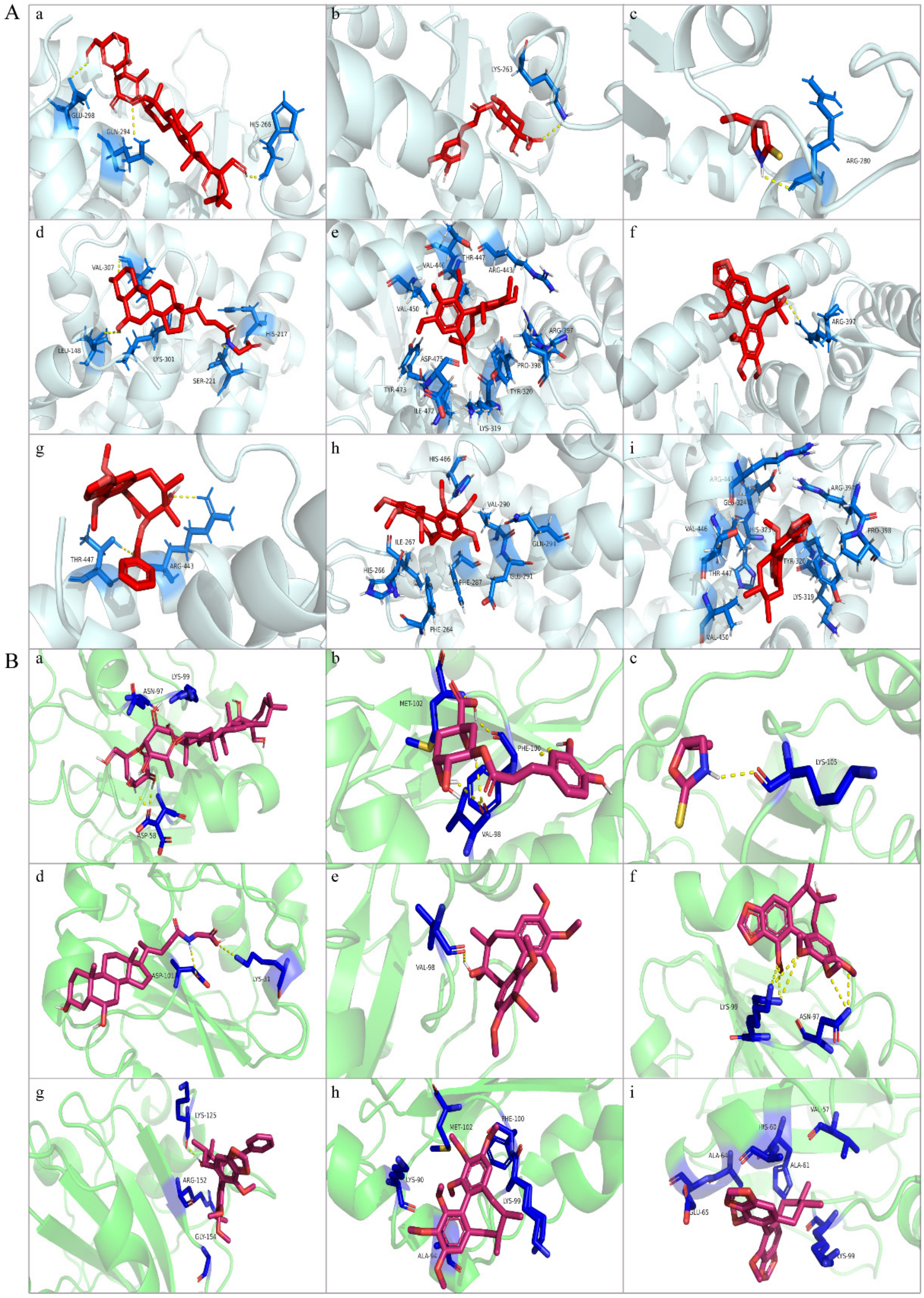

Figure S5. Predicted docking conformations between selected HGP quality markers and PPARγ/GPX4. A: Interactions between PPARγ and potential quality markers of HGP; **B**: Interactions between GPX4 and potential quality markers of HGP. (**a**: saikosaponin b2; **b**: chlorogenic acid; **c**: (R,S)-goitrin; **d**: glycohyodeoxycholic acid; **e**: schisandrin; **f**: schisandrol B; **g**: schisantherin A; **h**: schisandrin A; **i**: schisandrin C.).

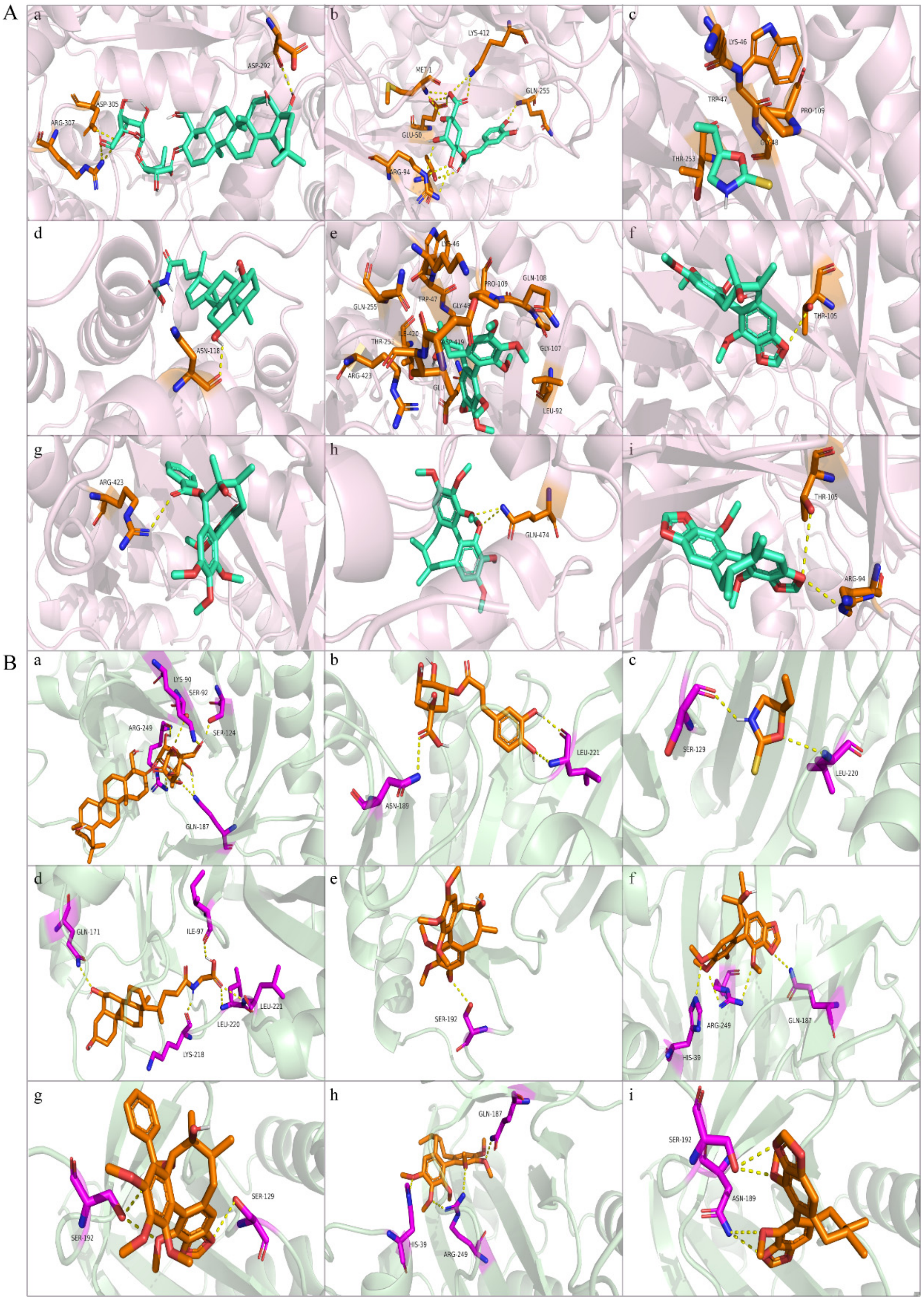

Figure S6. Predicted docking conformations between selected HGP quality markers and GCLC/GCLM. A: Interactions between GCLC and potential quality markers of HGP; **B**: Interactions between GCLM and potential quality markers of HGP. (**a**: saikosaponin b2; **b**: chlorogenic acid; **c**: (R,S)-goitrin; **d**: glycohyodeoxycholic acid; **e**: schisandrin; **f**: schisandrol B; **g**: schisantherin A; **h**: schisandrin A; **i**: schisandrin C.).

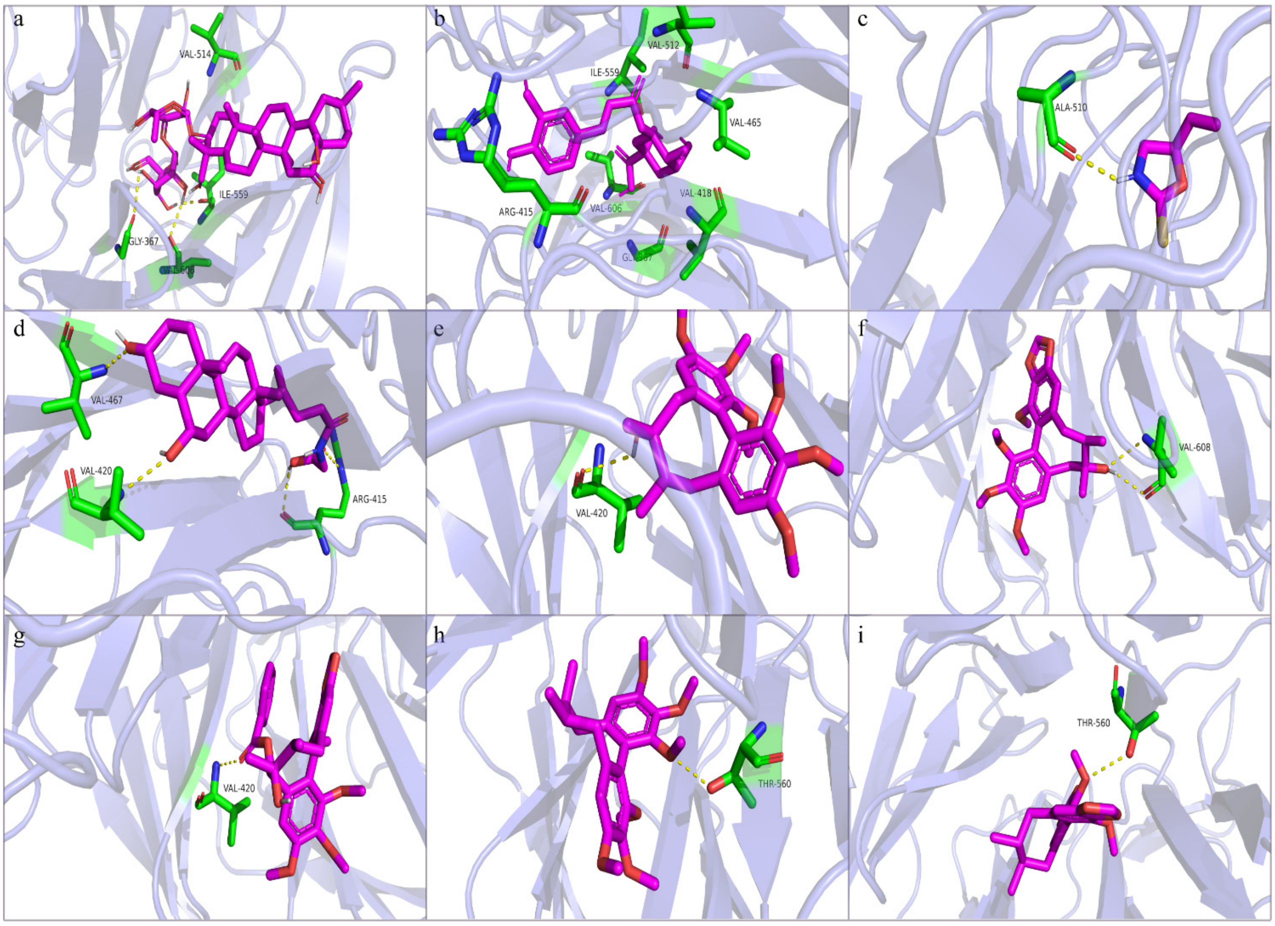

Figure S7. Predicted docking conformations between selected HGP quality markers and NRF2. (**a**: saikosaponin b2; **b**: chlorogenic acid; **c**: (R,S)-goitrin; **d**: glycohyodeoxycholic acid; **e**: schisandrin; **f**: schisandrol B; **g**: schisantherin A; **h**: schisandrin A; **i**: schisandrin C.).

Supplementary Tables

Table S1. Putatively annotated candidate metabolites showing counter-directional trends following HGP-H treatment in liver under positive ion mode.

No.	Metabolites	t_R/min	*m*/*z*	HMDB ID	Trends[a]	Trends[b]
1	Spermidine	1.039	146.16528	HMDB0001257	↓**	↑**
2	Ornithine	1.209	133.09740	HMDB0000214	↓***	↑**
3	Glycerophosphocholine	1.241	280.09180	HMDB0000086	↑**	↓
4	Glutamine	1.253	147.07643	HMDB0000641	↓*	↑*
5	Pyroglutamic acid	1.276	130.05013	HMDB0000267	↓***	↑*
6	Sarcosine	1.287	90.055070	HMDB0000271	↓***	↑*
7	Glutamic acid	1.289	148.06038	HMDB0000148	↓***	↑*
8	Argininic acid	1.291	176.10304	HMDB0003148	↓**	↑***
9	2-Phenylethylhexanoate	1.291	221.15387	HMDB0037718	↑***	↓
10	N-Acetylhistidine	1.303	198.08739	HMDB0032055	↓*	↑**
11	gamma-Aminobutyric acid	1.333	104.07078	HMDB0000112	↓***	↑*
12	L-Valine	1.336	118.08606	HMDB0000883	↓***	↑***
13	Deoxyguanosine	1.364	268.10455	HMDB0000085	↓*	↑
14	Niacinamide	1.39	123.05515	HMDB0001406	↑*	↓
15	5-aminovaleric acid betaine	1.791	160.13295	HMDB0240732	↓*	↑
16	Cysteinylglycine	1.794	179.04860	HMDB0000078	↓***	↑*
17	Glutamylcysteine	1.794	233.05972	HMDB0028816	↓***	↑*
18	Glutathione	1.794	308.09158	HMDB0000125	↓***	↑*

19	Guanosine monophosphate	1.8	364.06567	HMDB0001397	↓***	↑
20	Methionine	1.827	150.05832	HMDB0000696	↓***	↑*
21	S-Adenosylhomocysteine	1.959	385.12848	HMDB0000939	↓**	↑*
22	Uracil	1.964	113.03438	HMDB0000300	↓***	↑**
23	Guanine	2.412	152.05696	HMDB0000132	↓***	↑
24	Isoleucine	2.579	132.10210	HMDB0000172	↓***	↑*
25	2-Hydroxyisovalerylcarnitine	2.989	262.16556	HMDB0240421	↓**	↑*
26	Butyrylcarnitine	3.333	232.15501	HMDB0002013	↓*	↑
27	Tryptophan	3.43	205.09692	HMDB0000929	↓***	↑
28	(3R,7R)-1,3,7-Octanetriol	4.896	185.11505	HMDB0033625	↑***	↓*
29	Taurocholic acid	5.485	480.27859	HMDB0000036	↓*	↑*
30	O-Linoleoylcarnitine	7.767	424.34256	HMDB0240780	↓**	↑
31	LysoPC(16:1/0:0)	7.786	516.30573	HMDB0010383	↓***	↑
32	LysoPC(22:6(4Z,7Z,10Z,13Z,16Z,19Z)/0:0)	7.936	568.34052	HMDB0010404	↓**	↑*
33	LysoPC(0:0/18:2(9Z,12Z))	8.067	520.34070	HMDB0061700	↓*	↑
34	LysoPC(20:3(8Z,11Z,14Z)/0:0)	8.374	546.35614	HMDB0010394	↓***	↑
35	LysoPE(20:3(5Z,8Z,11Z)/0:0)	8.383	504.30743	HMDB0011515	↓***	↑
36	LysoPC(0:0/16:0)	8.565	496.33878	HMDB0240262	↓***	↑
37	LysoPC(18:3(9Z,12Z,15Z)/0:0)	8.565	518.32269	HMDB0010388	↓***	↑**
38	LysoPC(22:4(7Z,10Z,13Z,16Z)/0:0)	8.756	572.37091	HMDB0010401	↓***	↑
39	LysoPC(22:5(4Z,7Z,10Z,13Z,16Z)/0:0)	9.081	570.35321	HMDB0010402	↓***	↑*

40	LysoPC(P-18:0/0:0)	9.138	530.35828	HMDB0013122	↓**	↑
41	Arachidonic acid	11.169	305.24667	HMDB0001043	↓***	↑
42	Indole	11.416	118.06512	HMDB0000738	↑**	↓*

Note: Trends[a]: The change trend of the model group compared with the control group; Trends[b]: the change trend of HGP-H group compared with model group; ↓ indicates decreased relative abundance; ↑ indicates increased relative abundance; * indicates adjusted p value < 0.05, ** indicates adjusted p value < 0.01, *** indicates adjusted p value < 0.001.

Table S2. Putatively annotated candidate metabolites showing counter-directional trends following HGP-H treatment in liver under negative ion mode.

No.	Metabolites	t_R/min	*m*/*z*	HMDB ID	Trends[a]	Trends[b]
1	Aspartic acid	1.263	132.03014	HMDB0000191	↓**	↑**
2	Taurine	1.266	249.02249	HMDB0000251	↓**	↑
3	Glycerophosphoinositol	1.272	333.05884	HMDB0011649	↓**	↑
4	Uridine 5'-monophosphate	1.357	323.02893	HMDB0000288	↓***	↑
5	Malic acid	1.366	133.01401	HMDB0000156	↓**	↑*
6	4-Hydroxynicotinamide	1.923	137.03548	HMDB0246459	↑**	↓*
7	N-Acetyl-L-glutamic acid	2.048	188.05598	HMDB0001138	↓**	↑*
8	Succinic acid	2.232	117.01939	HMDB0000254	↓**	↑**
9	Inosine	2.461	267.07391	HMDB0000195	↓*	↑
10	2-Hydroxybutyric acid	2.865	103.04003	HMDB0000008	↓*	↑***
11	Adenylsuccinic acid	2.867	462.06778	HMDB0000536	↓**	↑
12	Xanthine	2.952	151.02568	HMDB0000292	↓*	↑*
13	Itaconic acid	3.216	129.01945	HMDB0002092	↓***	↑**
14	FAD	3.302	784.15106	HMDB0001248	↓**	↑
15	N-Acetyl-L-methionine	3.6	190.05408	HMDB0011745	↓***	↑*
16	Sulfolithocholyl glycine	5.131	512.26978	HMDB0002639	↓*	↑
17	LysoPE(0:0/24:6(6Z,9Z,12Z,15Z,18Z,21Z))	7.853	612.33014	HMDB0011499	↓**	↑
18	LysoPE(18:2(9Z,12Z)/0:0)	7.92	476.27682	HMDB0011507	↑**	↓
19	LysoPC(0:0/20:4(5Z,8Z,11Z,14Z))	7.944	588.33142	HMDB0061699	↓***	↑

20	LysoPE(20:4(5Z,8Z,11Z,14Z)/0:0)	7.95	500.27908	HMDB0011517	↓*	↑
21	LysoPE(22:4(7Z,10Z,13Z,16Z)/0:0)	8.018	528.30865	HMDB0011523	↓*	↑
22	LysoPE(16:0/0:0)	8.549	452.27853	HMDB0011503	↓*	↑
23	(8Z)-10-hydroxyhexadec-8-enoic acid	8.992	269.21222	HMDB0340885	↑*	↓
24	LysoPE(20:2(11Z,14Z)/0:0)	9.049	504.30930	HMDB0011513	↓***	↑
25	11-Hydroxyhexadecanoic acid	10.162	271.22800	HMDB0112189	↑***	↓

Note: Trends[a]: The change trend of the model group compared with the control group; Trends[b]: the change trend of HGP-H group compared with model group; ↓ indicates decreased relative abundance; ↑ indicates increased relative abundance; * indicates adjusted *p* value < 0.05, ** indicates adjusted *p* value < 0.01, *** indicates adjusted *p* value < 0.001.

Table S3. Putatively annotated candidate metabolites showing counter-directional trends following HGP-H treatment in serum under positive ion mode.

No.	Metabolites	t_R/min	*m*/*z*	HMDB ID	Trends[a]	Trends[b]
1	L-argininium(1+)	1.223	175.11891	HMDB0062762	↑**	↓***
2	(S)-carnitinium	1.317	162.11214	HMDB0062634	↑*	↓
3	gamma-Aminobutyric acid	1.318	104.07059	HMDB0000112	↓***	↑
4	Proline	1.366	116.07047	HMDB0000162	↓**	↑
5	Niacinamide	1.767	123.05511	HMDB0001406	↓***	↑***
6	Glycine	1.805	76.039270	HMDB0000123	↓**	↑
7	Methionine	1.863	150.05830	HMDB0000696	↓**	↑
8	Xanthine	1.87	153.04050	HMDB0000292	↓***	↑
9	L-Tyrosine	2.172	182.08089	HMDB0000158	↓***	↑*
10	(±)-Tryptophan	3.409	205.09682	HMDB0030396	↓**	↑**
11	Indoleacrylic acid	3.452	188.07034	HMDB0000734	↓**	↑**
12	Glutamylisoleucine	3.457	261.14413	HMDB0028822	↓***	↑
13	4-Hepteneoylglycine	4.441	186.11215	HMDB0094730	↑***	↓***
14	LysoPC(20:5(5Z,8Z,11Z,14Z,17Z)/0:0)	7.538	542.32281	HMDB0010397	↓***	↑**
15	LysoPE(22:6(4Z,7Z,10Z,13Z,16Z,19Z)/0:0)	7.898	526.29126	HMDB0011526	↓*	↑***
16	LysoPC(22:6(4Z,7Z,10Z,13Z,16Z,19Z)/0:0)	7.94	568.33868	HMDB0010404	↓**	↑*
17	LysoPE(20:4(8Z,11Z,14Z,17Z)/0:0)	7.993	502.29208	HMDB0011518	↓**	↑***
18	LysoPC(0:0/20:4(5Z,8Z,11Z,14Z))	8.011	544.33765	HMDB0061699	↓**	↑***
19	LysoPC(0:0/18:2(9Z,12Z))	8.056	542.32050	HMDB0061700	↑**	↓

20	LysoPE(16:0/0:0)	8.513	454.29251	HMDB0011503	↓***	↑***
21	11,12-Epoxyeicosatrienoic acid	8.563	303.23111	HMDB0004673	↓***	↑***
22	LysoPC(0:0/18:1(9Z))	8.785	522.35461	HMDB0061701	↓**	↑***
23	LysoPE(18:0/0:0)	9.983	482.32056	HMDB0011130	↓***	↑***
24	Phosphohydroxypyruvic acid	14.202	184.98537	HMDB0001024	↑***	↓*

Note: Trends[a]: The change trend of the model group compared with the control group; Trends[b]: the change trend of HGP-H group compared with model group; ↓ indicates decreased relative abundance; ↑ indicates increased relative abundance; * indicates adjusted p value < 0.05, ** indicates adjusted p value < 0.01, *** indicates adjusted p value < 0.001.

Table S4. Putatively annotated candidate metabolites showing counter-directional trends following HGP-H treatment in serum under negative ion mode.

No.	Metabolites	t_R/min	*m*/*z*	HMDB ID	Trends[a]	Trends[b]
1	Allantoin	1.342	157.03638	HMDB0000462	↓**	↑
2	2-Hydroxy-2-ethylsuccinic acid	2.407	161.04567	HMDB0059758	↓**	↑***
3	Inosine	2.455	267.07352	HMDB0000195	↓**	↑
4	Xanthosine	2.966	283.06787	HMDB0000299	↓***	↑
5	2-Hydroxy-3-methylbutyric acid	3.533	117.05566	HMDB0000407	↓**	↑***
6	Hippuric acid	3.948	178.05113	HMDB0000714	↑**	↓**
7	Indoxyl sulfate	3.95	212.00211	HMDB0000682	↑***	↓***
8	5-Hydroxyhexanoic acid	4.206	131.07146	HMDB0000525	↓***	↑***
9	5-Methoxyindoleacetate	4.829	204.06648	HMDB0004096	↑*	↓*
10	Chenodeoxycholyltaurine	5.471	498.29034	HMDB0242411	↑***	↓***
11	LysoPE(18:1(11Z)/0:0)	7.777	538.31580	HMDB0011505	↓**	↑***
12	LysoPE(22:6(4Z,7Z,10Z,13Z,16Z,19Z)/0:0)	7.914	524.27881	HMDB0011526	↓*	↑***
13	LysoPE(24:6(6Z,9Z,12Z,15Z,18Z,21Z)/0:0)	7.957	612.32953	HMDB0011529	↓***	↑***
14	LysoPE(20:4(5Z,8Z,11Z,14Z)/0:0)	7.976	500.27740	HMDB0011517	↓***	↑***
15	LysoPE(22:4(7Z,10Z,13Z,16Z)/0:0)	8.016	588.33142	HMDB0011523	↓***	↑***
16	8-HDoHE	8.423	343.22751	HMDB0060051	↓**	↑
17	LysoPC(15:0/0:0)	8.579	540.33203	HMDB0010381	↓*	↑***
18	12-Hydroxyarachidonic acid	8.583	319.22736	HMDB0060101	↓**	↑**
19	LysoPC(0:0/18:1(9Z))	8.796	566.34583	HMDB0061701	↓**	↑***

20	Linoleic acid	11.611	279.23267	HMDB0000673	↑**	↓**

Note: Trends[a]: The change trend of the model group compared with the control group; Trends[b]: the change trend of HGP-H group compared with model group; ↓ indicates decreased relative abundance; ↑ indicates increased relative abundance; * indicates adjusted p value < 0.05, ** indicates adjusted p value < 0.01, *** indicates adjusted p value < 0.001.

Table S5. RNA quality assessment results.

Sample	Concentration(ng/μL)	Total amount(μg)	OD260/280	OD260/230	RQN
Control1	586.82	20.54	1.91	2.22	8.9
Control2	563.3	19.72	1.94	2.14	9
Control3	474.43	16.6	1.95	2.16	8.7
Model1	294.02	10.29	1.94	2.19	8.8
Model2	366.41	12.82	1.96	2.16	8.9
Model3	370.66	12.97	1.94	2.21	8.7
HGP1	770.35	26.96	1.97	2.3	8.8
HGP2	581.67	20.36	1.92	2.1	9.1
HGP3	804.48	28.16	1.98	2.19	8.5

Table S6. Sequencing data quality control results.

Sample	Raw reads	Raw bases	Clean reads	Clean bases	Error rate (%)	Q20 (%)	Q30 (%)	GC content (%)
Control1	46971720	7092729720	46590808	6960679968	0.0121	98.67	95.81	47.32
Control2	40559562	6124493862	40209456	6000950615	0.0121	98.7	95.91	47.15
Control3	47252448	7135119648	46844168	6995683774	0.0122	98.61	95.63	48.53
Model1	40853262	6168842562	40496704	6053321568	0.0122	98.63	95.69	48.38
Model2	47417524	7160046124	46992632	7031830944	0.0122	98.6	95.63	48.1
Model3	45956934	6939497034	45546808	6803765731	0.0123	98.59	95.56	48.7
HGP1	46284980	6989031980	45864770	6857326276	0.0121	98.65	95.75	47.71
HGP2	40202016	6070504416	39855332	5943136469	0.0121	98.67	95.83	47.7
HGP3	47567270	7182657770	47192258	7047519034	0.0121	98.66	95.76	48.37

Raw reads: the total number of entries in the raw sequencing data; Raw bases: the total amount of raw sequencing data; Clean reads: the total number of sequencing data after quality control; Clean bases: the total amount of sequencing data after quality control; Error rate (%) : the average error rate of sequencing base corresponding to quality control data; Q20 (%) and Q30 (%) were used to evaluate the quality of sequencing data after quality control. Q20 and Q30 represent the percentages of bases with Phred quality scores of at least 20 and 30, respectively, corresponding to estimated base-call accuracies of 99% and 99.9%.

Table S7. Alignment statistics of clean reads against the reference genome.

Sample	Total reads	Total mapped	Multiple mapped	Unique mapped
Control1	46590808	43475380(93.31%)	6071480(13.03%)	37403900(80.28%)
Control2	40209456	37845999(94.12%)	5147979(12.8%)	32698020(81.32%)
Control3	46844168	45346758(96.8%)	4184503(8.93%)	41162255(87.87%)
Model1	40496704	39067718(96.47%)	3247280(8.02%)	35820438(88.45%)
Model2	46992632	45715143(97.28%)	3845811(8.18%)	41869332(89.1%)
Model3	45546808	44018576(96.64%)	3648686(8.01%)	40369890(88.63%)
HGP1	45864770	43980913(95.89%)	4669153(10.18%)	39311760(85.71%)
HGP2	39855332	38270617(96.02%)	4097338(10.28%)	34173279(85.74%)
HGP3	47192258	45460057(96.33%)	4439117(9.41%)	41020940(86.92%)

Total reads: the number of filtered sequences (i.e., Clean reads); Total mapped: the number and percentage of Clean reads that could be mapped to the genome; Multiple mapped: the number and percentage of Clean reads with multiple alignment positions on the reference sequence; Unique mapped: the number and percentage of Clean reads with unique alignment positions on the reference sequence.

Table S8. DEGs meeting the stringent criteria in both comparisons and showing opposite expression directions following HGP treatment.

Gene id	Gene name	Adjusted *p* value (HGP-H vs. Model)	Gene id	Gene name	Adjusted *p* value (HGP-H vs. Model)
ENSMUSG00000000078	*Klf6*	0.0252328889234	ENSMUSG00000038217	*Tlcd2*	1.60239479716E-6
ENSMUSG00000000275	*Trim25*	1.33906065352E-6	ENSMUSG00000038375	*Trp53inp2*	6.38278023917E-5
ENSMUSG00000000290	*Itgb2*	0.0334421341516	ENSMUSG00000038754	*Elovl3*	0.00109836611017
ENSMUSG00000000876	*Pxmp4*	0.0056629631174	ENSMUSG00000039087	*Rreb1*	0.00259957828273
ENSMUSG00000001143	*Lman2l*	0.0378560083838	ENSMUSG00000039220	*Ppp1r10*	2.84398257471E-13
ENSMUSG00000002289	*Angptl4*	0.00126189502987	ENSMUSG00000039457	*Ppl*	0.0290208274357
ENSMUSG00000002346	*Slc25a42*	1.87868666817E-9	ENSMUSG00000039533	*Mmd2*	8.88942595166E-5
ENSMUSG00000003134	*Tbc1d8*	0.0278739022158	ENSMUSG00000039745	*Htatip2*	1.34332259604E-8
ENSMUSG00000003849	*Nqo1*	2.10562305854E-6	ENSMUSG00000040446	*Rprd1a*	0.021964
ENSMUSG00000003948	*Mmd*	3.19500190823E-7	ENSMUSG00000040616	*Tmem51*	0.0483545037416
ENSMUSG00000006445	*Epha2*	0.00386230132722	ENSMUSG00000040660	*Cyp2b9*	0.0281849051199
ENSMUSG00000008153	*Clstn3*	2.73854543673E-7	ENSMUSG00000041920	*Slc16a6*	0.00264151059086

ENSMUSG00000009378	*Slc16a12*	0.000811902701309	ENSMUSG00000042349	*Ikbke*	0.00222843245119
ENSMUSG00000009633	*G0s2*	2.36850080695E-26	ENSMUSG00000042622	*Maff*	1.32348001563E-6
ENSMUSG00000010051	*Hyal1*	0.00153833360735	ENSMUSG00000044367	*Slc16a13*	0.000388218676799
ENSMUSG00000011305	*Plin5*	0.00225182150426	ENSMUSG00000044469	*Tnfaip8l1*	2.79839497621E-5
ENSMUSG00000015357	*Clpx*	0.0149690627142	ENSMUSG00000044534	*Ackr2*	0.0281849051199
ENSMUSG00000015568	*Lpl*	0.00804465971517	ENSMUSG00000046876	*Atxn1*	0.017247
ENSMUSG00000018900	*Slc22a5*	2.93288382085E-8	ENSMUSG00000046947	*Adck2*	0.0414678900554
ENSMUSG00000019082	*Slc25a22*	1.02304840336E-10	ENSMUSG00000047492	*Inhbe*	1.07647395661E-8
ENSMUSG00000019122	*Ccl9*	6.8484591507E-5	ENSMUSG00000048307	*Ankrd46*	0.00556746793549
ENSMUSG00000019935	*Slc17a8*	1.13751037562E-12	ENSMUSG00000048371	*Pdp2*	0.000286414504659
ENSMUSG00000020000	*Moxd1*	0.0366553320432	ENSMUSG00000048486	*Fitm2*	1.07647395661E-8
ENSMUSG00000020614	*Fam20a*	0.00107329233333	ENSMUSG00000048782	*Insc*	0.0442200582202
ENSMUSG00000020641	*Rsad2*	0.0131264266159	ENSMUSG00000049502	*Dtx3l*	0.000607174617209
ENSMUSG00000020777	*Acox1*	2.92759994637E-5	ENSMUSG00000049723	*Mmp12*	2.4954030103E-12
ENSMUSG00000021244	*Ylpm1*	0.0178564346878	ENSMUSG00000050335	*Lgals3*	0.000167589094911

ENSMUSG00000021400	*Wrnip1*	0.00623841763283	ENSMUSG00000050860	*Phospho1*	1.55660575056E-9
ENSMUSG00000021947	*Cryl1*	0.00605834030523	ENSMUSG00000051000	*Fhip1a*	0.0283217693932
ENSMUSG00000022041	*Chrna2*	8.70022454948E-6	ENSMUSG00000051427	*Ccdc157*	0.00243092880111
ENSMUSG00000022094	*Slc39a14*	6.24859575024E-5	ENSMUSG00000051483	*Cbr1*	0.000728265701772
ENSMUSG00000022237	*Ankrd33b*	0.00461085083966	ENSMUSG00000052305	*Hbb-bs*	1.53727527924E-5
ENSMUSG00000022615	*Tymp*	0.0126776226403	ENSMUSG00000052632	*Asap2*	0.0108389870506
ENSMUSG00000022676	*Snai2*	0.0176947760763	ENSMUSG00000052928	*Ctif*	0.000296798224006
ENSMUSG00000022816	*Fstl1*	0.0297348765225	ENSMUSG00000053559	*Smagp*	0.0144544987054
ENSMUSG00000022885	*St6gal1*	0.000607174617209	ENSMUSG00000055065	*Ddx17*	0.0394216133764
ENSMUSG00000023034	*Nr4a1*	4.79168747181E-5	ENSMUSG00000056035	*Cyp3a11*	2.67363351138E-13
ENSMUSG00000023044	*Csad*	0.00659274162741	ENSMUSG00000056429	*Tgoln1*	0.00623841763283
ENSMUSG00000023913	*Pla2g7*	0.0279964711316	ENSMUSG00000057110	*Cntrl*	0.0393216288417
ENSMUSG00000023963	*Cyp39a1*	2.50992052902E-15	ENSMUSG00000057342	*Sphk2*	0.0161361332233
ENSMUSG00000024029	*Tff3*	0.0342773417479	ENSMUSG00000057778	*Cyb5d2*	0.00647013798658
ENSMUSG00000024277	*Mapre2*	0.000811902701309	ENSMUSG00000058135	*Gstm1*	5.30150277974E-5

ENSMUSG00000024589	*Nedd4l*	0.00196476182733	ENSMUSG00000060519	*Tor3a*	4.75047173857E-6
ENSMUSG00000024818	*Slc25a45*	0.0197376018709	ENSMUSG00000060961	*Slc4a4*	1.72130488811E-5
ENSMUSG00000024987	*Cyp26a1*	0.0278739022158	ENSMUSG00000061292	*Cyp3a59*	9.68239992476E-21
ENSMUSG00000025002	*Cyp2c55*	3.5908410717E-5	ENSMUSG00000061313	*Ddhd2*	0.00335923727452
ENSMUSG00000025375	*Aatk*	0.00282048083594	ENSMUSG00000061780	*Cfd*	4.25017554716E-58
ENSMUSG00000025405	*Inhbc*	0.0355319550721	ENSMUSG00000063354	*Slc39a4*	5.46198169462E-11
ENSMUSG00000025504	*Eps8l2*	3.09029116841E-5	ENSMUSG00000063590	*Slc22a28*	0.00548980809181
ENSMUSG00000026639	*Lamb3*	8.74220047797E-8	ENSMUSG00000064225	*Paqr9*	2.19570855969E-6
ENSMUSG00000026728	*Vim*	0.0411117752015	ENSMUSG00000067144	*Slc22a7*	4.14613538728E-7
ENSMUSG00000026819	*Slc25a25*	4.70645393584E-15	ENSMUSG00000069516	*Lyz2*	0.0107315095053
ENSMUSG00000026822	*Lcn2*	0.00226874204546	ENSMUSG00000069917	*Hba-a2*	2.92759994637E-5
ENSMUSG00000026827	*Gpd2*	0.00270845234319	ENSMUSG00000069919	*Hba-a1*	1.87357722254E-5
ENSMUSG00000026853	*Crat*	0.000306690934287	ENSMUSG00000070392	*Gm20634*	0.00679288431905
ENSMUSG00000026922	*Agpat2*	0.0113417177822	ENSMUSG00000071551	*Akr1c19*	2.71196581852E-11
ENSMUSG00000027274	*Mkks*	0.0445141404819	ENSMUSG00000072676	*Tmem254*	8.70022454948E-6

ENSMUSG00000027502	*Rtf2*	0.000728265701772	ENSMUSG00000072949	*Acot1*	0.0127979756076
ENSMUSG00000027580	*Helz2*	0.00193792962745	ENSMUSG00000073555	*Iigp1c*	0.0297299939217
ENSMUSG00000027762	*Sucnr1*	0.0281849051199	ENSMUSG00000073940	*Hbb-bt*	0.000182094031295
ENSMUSG00000028195	*Ccn1*	0.000513174489191	ENSMUSG00000074254	*Cyp2a4*	0.0294646311844
ENSMUSG00000028494	*Plin2*	9.74906918851E-11	ENSMUSG00000075551	*Cyp3a41a*	0.0182102780438
ENSMUSG00000028538	*St3gal3*	2.74849468604E-5	ENSMUSG00000078143	*Gm17344*	5.12519528759E-5
ENSMUSG00000028655	*Mfsd2a*	0.00220358459702	ENSMUSG00000078650	*G6pc*	1.34232036933E-7
ENSMUSG00000028967	*Errfi1*	0.0429823625633	ENSMUSG00000078866	*Zfp970*	0.0233028802617
ENSMUSG00000029816	*Gpnmb*	1.32093899204E-6	ENSMUSG00000079164	*Tlr5*	0.000357207488704
ENSMUSG00000030168	*Adipor2*	2.85889923455E-6	ENSMUSG00000079293	*Clec7a*	1.13149557278E-5
ENSMUSG00000030256	*Bhlhe41*	0.0134391940852	ENSMUSG00000079334	*Naa80*	0.00367702277607
ENSMUSG00000030545	*Pex11a*	0.000679056907355	ENSMUSG00000085148	*Mir22hg*	0.00511370995241
ENSMUSG00000030643	*Rab30*	1.13149557278E-5	ENSMUSG00000085995	*Gm2788*	0.00748356706561
ENSMUSG00000030762	*Aqp8*	0.0136822687919	ENSMUSG00000087579	*Hectd2os*	8.70221230663E-6
ENSMUSG00000030849	*Fgfr2*	0.0414678900554	ENSMUSG00000090035	*Galnt4*	0.0278739022158

ENSMUSG00000031024	*Dennd2b*	7.67890537575E-7	ENSMUSG00000090175	*Ugt1a9*	2.54270056518E-28
ENSMUSG00000031327	*Chic1*	0.0144918129734	ENSMUSG00000091780	*Sco2*	1.57953763036E-5
ENSMUSG00000031379	*Pir*	0.0135692171113	ENSMUSG00000097290	*1300002E11Rik*	0.0238510690628
ENSMUSG00000031775	*Pllp*	0.0414678900554	ENSMUSG00000097691	*9030616G12Rik*	0.00946781812235
ENSMUSG00000032348	*Gsta4*	3.73651957749E-5	ENSMUSG00000097730	*Gm26588*	0.0016759461366
ENSMUSG00000032349	*Elovl5*	3.43020514937E-10	ENSMUSG00000101517	*4732465J04Rik*	0.00710411322757
ENSMUSG00000032724	*Abtb2*	0.0176502809467	ENSMUSG00000104348	*Gm37691*	1.07647395661E-8
ENSMUSG00000032849	*Abcc4*	2.31690173418E-5	ENSMUSG00000106040	*Cyp3a63-ps*	0.0253053028227
ENSMUSG00000032902	*Slc16a1*	0.000607370399609	ENSMUSG00000110035	*Gm30931*	0.0446820571634
ENSMUSG00000032946	*Rasgrp2*	0.0391984133447	ENSMUSG00000110827	*Gm32281*	0.0398697758199
ENSMUSG00000034248	*Slc25a37*	6.5453311496E-10	ENSMUSG00000111709	*Gm3776*	0.0229078134577
ENSMUSG00000036957	*Lrfn3*	0.0121610454971	ENSMUSG00000112527	*Gm35696*	0.00271343322957
ENSMUSG00000037348	*Paqr7*	9.36317531203E-8	ENSMUSG00000112984	*Gm48419*	0.00145685144303
ENSMUSG00000037379	*Spon2*	2.37635253556E-8	ENSMUSG00000113050	*Gm8016*	0.018103
ENSMUSG00000037440	*Vnn1*	0.00372253996142	ENSMUSG00000115708	*Gm49187*	0.00281869129071

ENSMUSG00000037905	*Bri3bp*	0.00467911053192	ENSMUSG00000115867	*Gm17753*	0.0102015232617
ENSMUSG00000038147	*Cd84*	0.042644	ENSMUSG00000117485	*Gm19696*	0.00226874204546
ENSMUSG00000120139	*Gm32342*	0.00486995274209	ENSMUSG00000119973	*Gm30842*	0.0223449309074
ENSMUSG00000121092	*Gm56862*	0.00231259036957			

All listed genes met an adjusted p value < 0.05 and $|\log2FoldChange| > 0.585$ in both the Model versus Control and HGP versus Model comparisons. Among these genes, 51 were increased in the Model versus Control comparison and decreased in the HGP versus Model comparison, whereas 130 showed the opposite pattern. The adjusted p values presented in the table correspond to the HGP-H versus Model comparison.

Table S9. Predicted binding energy (kcal/mol) of nine potential HGP quality markers with selected proteins.

Component	GPX4	PPARγ	GCLM	GCLC	NRF2
saikosaponin b2	-7.6	-7.7	-8.4	-8.4	-9.9
chlorogenic acid	-6.9	-8.2	-7	-7.4	-9.6
(*R,S*)-goitrin	-3.8	-4.3	-3.9	-3.9	-4.8
glycohyodeoxycholic acid	-5.9	-5.9	-6.2	-6.3	-6.4
schisandrin	-6.7	-7.2	-6.8	-7.3	-7.4
schisandrol B	-6.6	-6.6	-7.3	-7.8	-7.7
schisantherin A	-6.1	-5.8	-6.6	-6.5	-6.7
schisandrin A	-7	-7.8	-7.6	-8.2	-7.7
schisandrin C	-7.7	-7.7	-7.5	-8	-10.1
